# A Middle Stone Age occupation identified at Baden-Baden in the grasslands of the Free State, South Africa

**DOI:** 10.1038/s41598-026-43246-9

**Published:** 2026-04-06

**Authors:** Maïlys Richard, Beatrice Bin, Benoit Longet, Michaela Ecker, Nils Andersen, Will Archer, Myra Gohodzi, Sharon Holt, C. Britt Bousman, Michael B. Toffolo

**Affiliations:** 1 Archéosciences Bordeaux, UMR 6034 CNRS-Bordeaux Montaigne University, Esplanade des Antilles, Pessac, 33607 France; 2 Department of Early Prehistory and Quaternary Ecology, University of Tübingen, Burgsteige 11, 72070 Tübingen, Germany; 3 Geochronology and Geology Programme, National Research Centre on Human Evolution (CENIEH), Paseo Sierra de Atapuerca 3, Burgos, 09002 Spain; 4 Institute of Prehistoric and Protohistoric Archaeology, Kiel University, Johanna- Mestorf-Straße 2-6, 24118 Kiel, Germany; 5 Archaeology Department, McGregor Museum, Atlas Street, Kimberley, 8301 South Africa; 6 Leibniz Laboratory for Radiometric Dating and Isotope Research, Kiel University, Max-Eyth-Straße 11-13, 24118 Kiel, Germany; 7 Department of Archaeology and Anthropology, Max Planck Partner Group, National Museum Bloemfontein, Bloemfontein, 9301 South Africa; 8 Florisbad Quaternary Research Station, National Museum Bloemfontein, Bloemfontein, 9301 South Africa; 9 Department of Geology, University of the Free State, Zastron Street, Bloemfontein, 9301 South Africa; 10 Department of Anthropology, The George Washington University, 2110 G St. NW, Washington, DC 20052 USA; 11 Department of Anthropology, Texas State University, 601 University Drive, San Marcos, TX 78666-4684 USA; 12 Department of Plant Sciences, University of the Free State, Zastron Street, Bloemfontein, 9301 South Africa

**Keywords:** Middle Stone Age, Free State, South Africa, Luminescence, Bioturbation, Finite Mixture Model, Ecology, Ecology, Environmental sciences, Solid Earth sciences

## Abstract

In southern Africa, Marine Isotope Stage (MIS) 5 (~ 130 − 71 ka) is a crucial period in the evolution of *Homo sapiens* but poorly documented in the central interior of the subcontinent. We report here results from Baden-Baden 2, a newly discovered open-air site in the western Free State grasslands of South Africa that documents Middle Stone Age occupations dated to MIS 5. The lithic assemblage differs from known MIS 5 and post-MIS 5 industries and shows similarities with industries typically dated to MIS 6 (~ 191 − 130 ka). In order to geochronologically constrain the occupation of Baden-Baden 2, sediments for optically stimulated luminescence (OSL) dating were collected together with micromorphology blocks to correlate formation processes and luminescence data. Magnetic susceptibility and plant biomarker analyses were used to identify changes in sediment supply and reconstruct past environments, revealing a shift in sediment source or climate regime during the period of human occupation, which took place between 91 ± 8 ka and 75 ± 7 ka. The palaeoenvironment shows three relatively stable phases that match the three broad phases of sediment input identified. The combination of OSL and micromorphology shows extensive bioturbation due to termite activity in the whole sequence. We discuss here the issue of dating quartz grains in bioturbated contexts, using single grain analyses combined with the Finite Mixture Model. Our results suggest that sand at the site was deposited from 106 ± 8 ka, for the base of the sequence, to 120 ± 30 a, for the modern sample at the top. The Baden-Baden 2 assemblage overlaps temporally with different industries in other South African regions, underscoring the need for more systematic investigation of MIS 5 in the interior of South Africa.

**Supplementary Information:**

The online version contains supplementary material available at 10.1038/s41598-026-43246-9.

## Introduction

The emergence of *Homo sapiens* in Africa during the late Middle Pleistocene overlaps with the onset of the Middle Stone Age (MSA), which is characterised by numerous innovations, especially with regard to lithic technology^1–3^. In southern Africa, these innovations become particularly apparent starting from 100,000 years ago^4^, during Marine Isotope Stage (MIS) 5 (~ 130 − 71 ka^5^), and represent a major increase in cognitive complexity in *H. sapiens* groups^6^. Most of the evidence comes from coastal sites in South Africa, although renewed research in the interior of the subcontinent has highlighted the important role of the Savanna and Grassland Biomes^7^ in the evolution of early *H. sapiens*^8–11^. In particular, during MIS 5 the central interior plateau of South Africa was characterised by an extensive system of rivers and lakes that supported human groups and large animal populations in an otherwise semi-arid ecosystem^12–14^. This favourable setting likely promoted human settlement of the open landscapes typical of the grasslands, especially around permanent water bodies^15–17^. While a few major MIS 5 sites are known in the central interior, the overall paucity of stratified occurrences accurately dated to MIS 5 hinders our understanding of lithic technology and population expansion in this vast swathe of land.

The vast majority of the ages that frame MSA occupations in South Africa have been obtained using trapped-charge methods, and mainly by optically stimulated luminescence (OSL) of quartz grains^18–24^. This method is particularly efficient in the interior of South Africa because Kalahari sands are characterised by a remarkably bright signal and thus can be considered as good candidates for OSL dating^20,25^. However, the application of OSL is often limited by preservation issues, especially bioturbation that causes mixing of the quartz grains within or between archaeological layers^26^, and/or partial bleaching of the dated grains^19^. This becomes especially apparent at open-air sites, which are prone to post-depositional alteration^25,27^. These problems are further compounded by the scarce application of micro-contextual characterisation methods, such as micromorphology of sediments and magnetic susceptibility, which can improve the interpretation of OSL ages by identifying post-depositional processes and breaks in the sedimentation, respectively^26,28,29^. Therefore, overcoming these issues is of fundamental importance to define the chronology of MIS 5 lithic assemblages, which will eventually allow a more informed narrative of the evolution of *H. sapiens* in southern Africa^8,30–32^.

Here we present results from Baden-Baden 2, a newly discovered, open-air MSA site located in the grasslands of the western Free State Province (henceforth Free State) of South Africa. Using a multi-analytical approach including micromorphology of sediments, infrared spectroscopy, magnetic susceptibility, characterisation of plant biomarkers, and OSL dating of quartz, we were able to identify an intact archaeological context and to determine its palaeoenvironment and age, which falls within MIS 5. Despite its age, the lithic assemblage shows broad technological similarities with assemblages often grouped under the label early Middle Stone Age (EMSA), particularly Florisbad, which remains the only EMSA assemblage of this general character in the Free State^15,33,34^. The results obtained provide a new MIS 5 reference for the Free State that complements the available information from Florisbad, and represent the first Pleistocene plant biomarker study in the Grassland Biome of South Africa. We then discuss the OSL ages of Baden-Baden 2 within the broader context of the lithic industries of MIS 8 − 4 (~ 300 − 57 ka^5^) in South Africa and show how they contribute to the refinement of the chronological framework of the interior grasslands during MIS 5.

### Baden-Baden 2

**Fig. 1:**
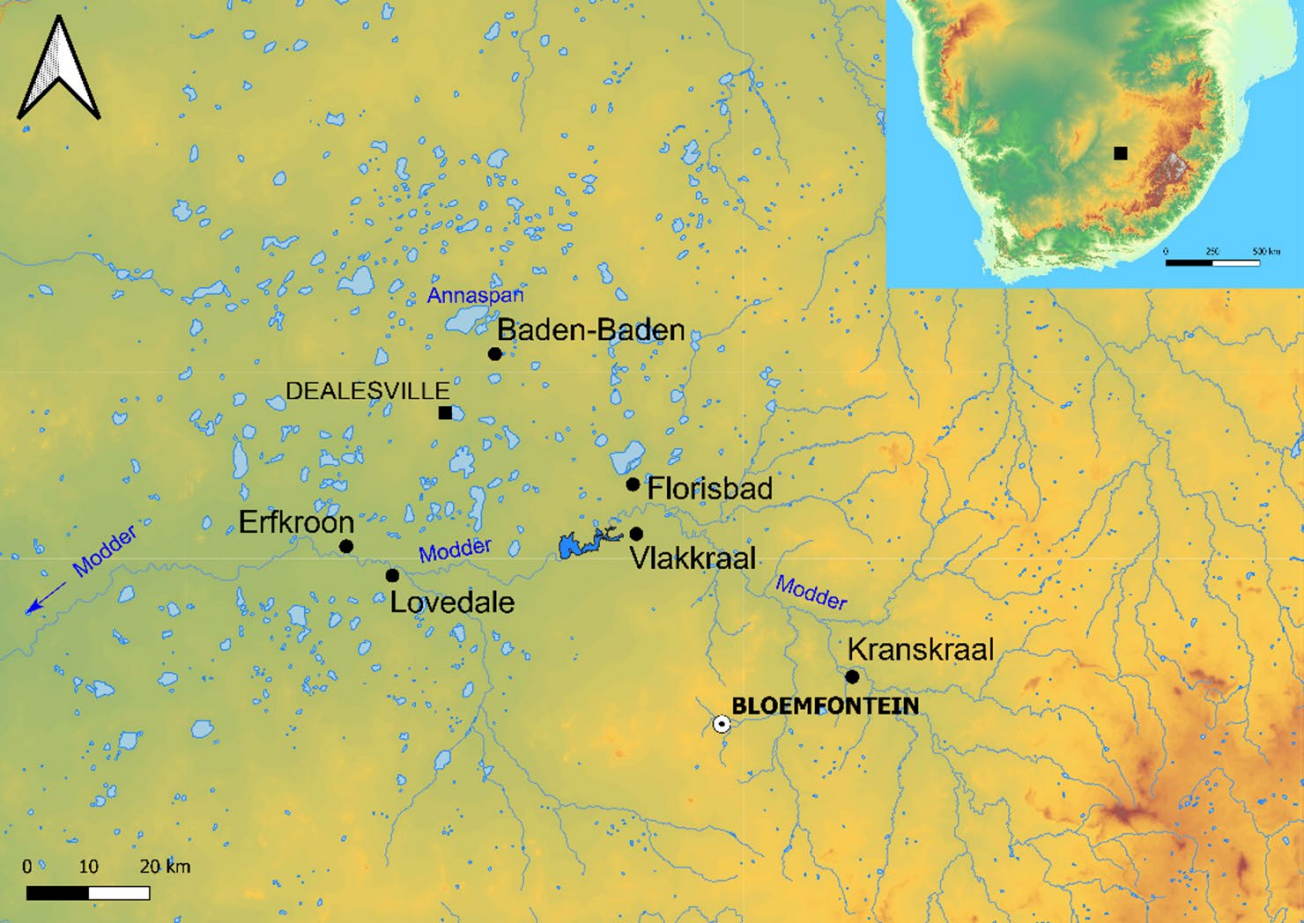
Map of the western Free State showing the location of Baden-Baden and other major Pleistocene sites in the region. Pale-blue features represent pans. Source: Shuttle Radar Topography Mission 1-arc second Global, National Geospatial-Intelligence Agency (NGA), USGS Earth Resources Observation and Science (EROS), retrieved from NASA Earthdata.

**Fig. 2:**
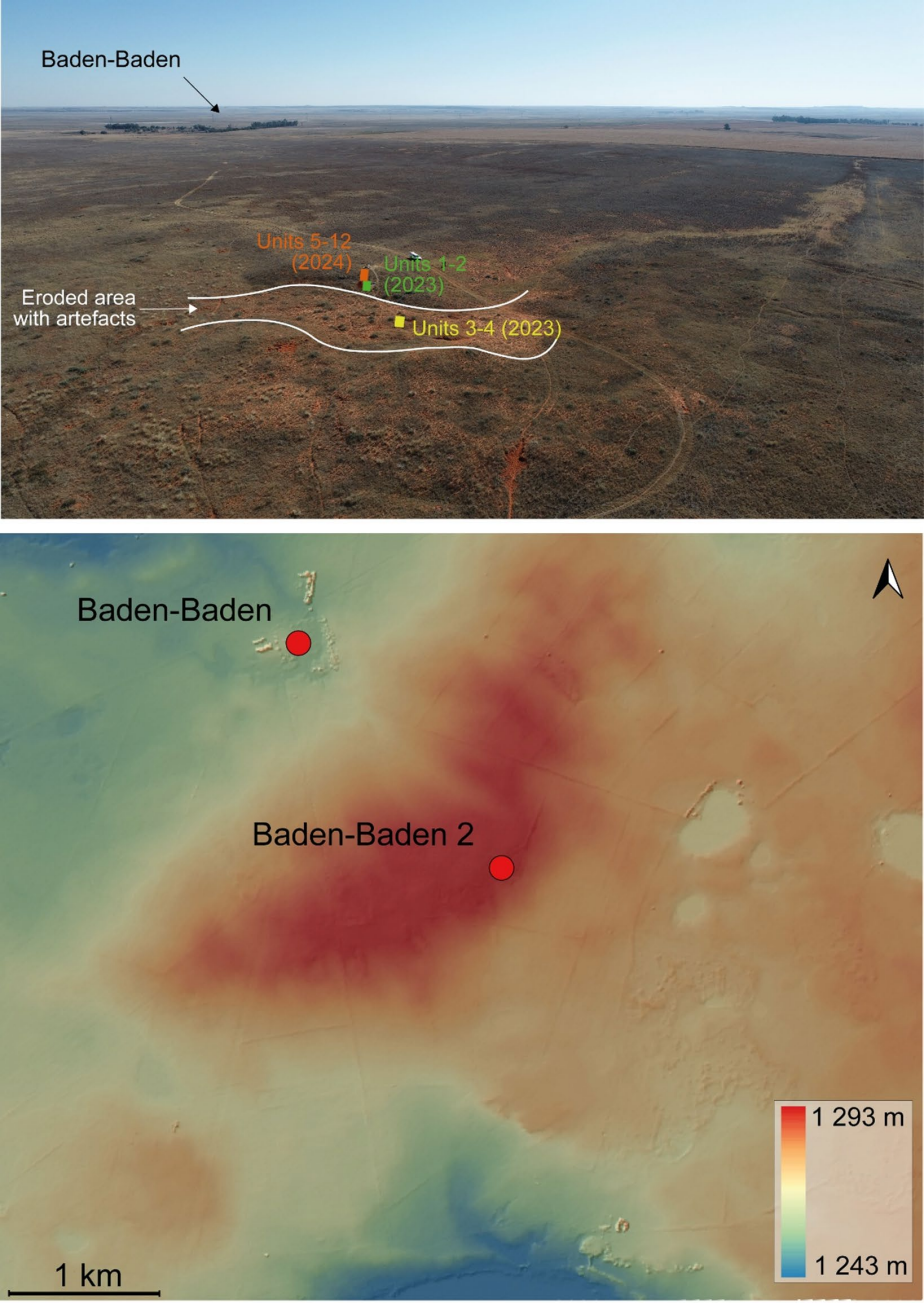
Location of Baden-Baden 2. Top: aerial view of the site showing the eroded area with surface artefacts and the 2023 and 2024 excavation trenches; Baden-Baden (LSA site) is marked by a line of trees in the background. Bottom: digital surface model (50 cm resolution) of the Baden-Baden farm showing the location of Baden-Baden and Baden-Baden 2 (pale yellow and blue patches represent pans). The Annaspan lunette dune appears in red.

Baden-Baden is a farm located 1 km to the southeast of the Annaspan seasonal lake, one of the largest saltpans of the Free State, near Dealesville, within the Dry Highveld Grassland Bioregion of the Grassland Biome^7^(Fig. 1). The farm is archaeologically known for a spring site that produced Late Pleistocene sediments and faunal remains, and a Holocene Later Stone Age (LSA) occupation^35^. A scatter of lithic artefacts (28°35’17.79"S 25°50’40.56"E) was discovered by the landowner 2 km to the southeast of the LSA site, at the top of a large dune covered in grass that is part of a system of lunette dunes on the southeastern margin of Annaspan. At 1300 m a.s.l., the dune represents the highest point between Annaspan and the Modder River, located 35 km to the south, and overlooks a valley featuring several pans organised in a decreasing gradient until at least Dealesville (Fig. 2). These pans are thought to be the remnant of ancient drainages disrupted by tectonic uplift^36–38^. The dune was surveyed in February 2023 to determine the broad archaeological period of the surface scatter (Earlier Stone Age—ESA, MSA, or LSA) and assess its potential for excavation. The artefacts were concentrated in a shallow depression and appeared to erode out of the southern side of the dune, below its top (Fig. 2). In addition, the scatter stretched in an east-west direction following several paths carved by cattle. Artefacts showed traits consistent with MSA technology, although it was not possible to assign them to a specific technocomplex based on cursory inspection. The site was called Baden-Baden 2 to distinguish it from the LSA spring site on the same property. During two excavation seasons in 2023 and 2024, sediment samples for OSL dating were collected together with micromorphology blocks to assess the extent of the post-depositional processes that may affect the ages, mainly bioturbation related to the activity of termites. Samples for magnetic susceptibility were also taken to search for changes in sediment supply, as well as for plant biomarkers to reconstruct palaeoenvironments.

### Dating bioturbated open-air sites using single grains of quartz

Framing human occupation in bioturbated contexts can be extremely complex since quartz grains are carried by termites from one layer to the other and/or moved through the channels dug by these insects and by plant roots. Many studies have pointed out the difficulties in dating such mixed and perturbated contexts^39,40^. To address this issue, single-grain analyses must be conducted to assess the extent of the mixing, and to apply statistical models. For such contexts, the Finite Mixture Model^41,42^ can be used combined with single-grain analyses. It allows identifying a number of discrete *k* components among the grain populations. Each of these components has a mean equivalent dose (D_e_) and takes into account the same common dispersion parameter, σ_b_. A maximum log likelihood (llik) and the Bayes Information Criterion (BIC) are calculated, the optimal combination being the maximum llik with the lowest BIC score. The BIC will decrease as the number of components is added, but will increase when the extra components are not necessary^43^. The number of components that gives the lowest BIC score is thus advised to be used for the age calculation^44^. The doses and relative proportion of these components are given by the model, and the dose component with the highest proportion is often taken as the most representative^39,45,46^, although in some case where extensive bioturbation led to the introduction of a high proportion of young grains, the oldest component may be selected to calculate ages^25^.

However, the results of the FMM are dependent on the value of σ_b_ used^47^. Some authors used a fixed value (i.e., generally 0.15–0.20) based on the mean overdispersion (OD) value observed in well-bleached and undisturbed samples measured using single grains (see for example Table 4 in Arnold and Roberts^44)^; nevertheless, the OD may vary from sample to sample^44^. Others used an alternative approach that implies varying the σ_b_ to fit the FMM, and select the best fit using the BIC criteria^48,49^. Tribolo et al.^39^ observed that age results calculated using the main component were comparable at one sigma regardless of which approach was used, fixed σ_b_ at 0.15 or using the value of σ_b_ that gives the best max llik and BIC combination. Here we ran the FMM by varying the σ_b_ value, and selected the optimal combination of σ_b_ and *k* that gave the lower BIC score to calculate our ages. We compare our results with those obtained using a fixed value of 0.20 and discuss the relevance of each approach and the consequences on the age interpretation.

## Results

### Geoarchaeology

**Fig. 3:**
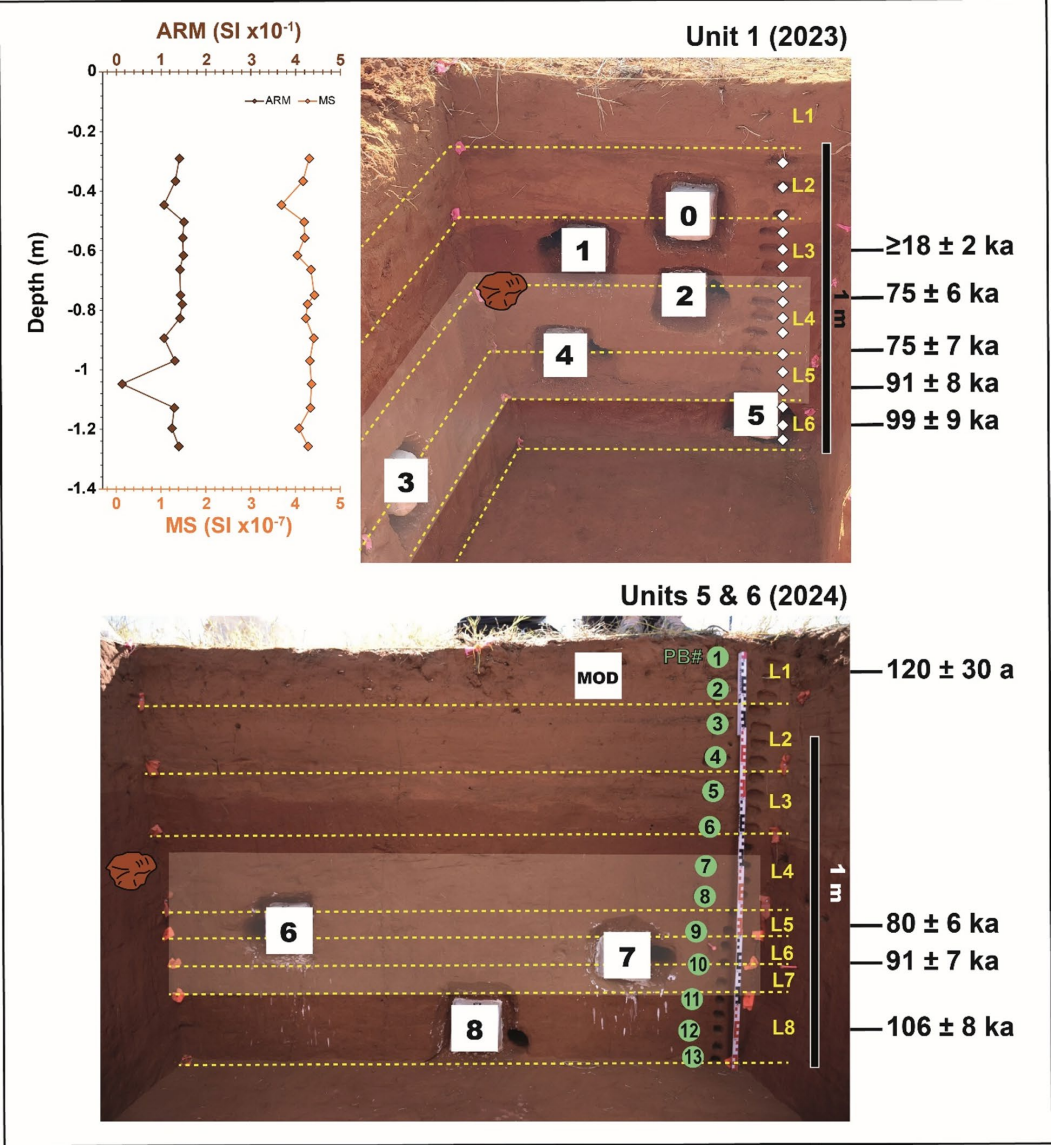
North sections of Units 1, 5, and 6 showing the location of sediment samples for OSL (1–8, modern), micromorphology (0–8), and plant biomarkers (1–13). Note that squares mark the combined OSL, gamma spectrometry, and micromorphology hollows left after sample collection; OSL sample depths are reported in Table 1. L: level; PB: plant biomarkers; MOD: modern. The levels are arbitrary and are 20 cm thick except for L5 to L7 in 2024, which are 10 cm thick. The first archaeological level encountered in each trench is marked by an artefact. The archaeological levels are marked by shaded areas. The dating samples are numbered from the younger to the older, i.e., from 1 to 5 for Unit 1 and from 6 to 8 for Units 5 & 6. The age of sample BAD1 may only represent a minimum estimate. The inset shows the magnetic susceptibility (MS) and anhysteretic remanent magnetisation (ARM) plots spanning Levels 2–6 in Unit 1, adjusted to match the MS and ARM samples shown in the section (diamonds). The ages are given in thousand years (ka) for the archaeological samples and in years (a) for the modern one.

The excavation of the site allowed to identify two layers of sediment separated by a sharp boundary, which we called orange sand and red sand, from top to bottom (Fig. 3). In Units 1–2 and 5–12 (Supplementary Figure S1-S5), artefacts appeared at ~ 60 cm depth within the red sand, and until ~ 110 cm depth. At ~ 95–100 cm depth, artefacts were found lying flat in clusters punctuating the excavation trenches, suggesting the existence of an occupation surface (Supplementary Figure S6). In Units 3–4, in the eroded area littered with surface artefacts, lithics appeared throughout the orange sand and until the contact between orange and red sand (Supplementary Figure S7). Lithics in the orange sand were similar in technology and patination to those found in the red sand of Units 1–2 and 5–12, often showing fresh breaks and embedded in thin beds of modern sheetwash, from which we deduced their secondary deposition caused by erosion from the southern flank of the dune and trampling and transport by roaming cattle.

Fourier transform infrared spectroscopy (FTIR) shows that the orange sand comprises a large proportion of quartz (bands at 1087 − 1080, 798, 779, 695, 513, and 463 cm^− 1^) and a much smaller amount of clay minerals of the smectite (3621, 1040 cm^− 1^) and kaolinite (3696, 915 cm^− 1^) groups. In the red sand, the proportion of clay minerals increases significantly, although quartz remains the major component (Supplementary Figure S8). Image analysis shows that this quartz falls mainly within the fine sand fraction and is characterised by high sensitivity circularity (HSC) mean values ranging from 0.826 to 0.859, indicating a relatively high degree of roundness. Similarly, the mean HSC values of the 100–120 μm fraction used for OSL dating range from 0.828 to 0.866 (Supplementary Figures S9-S10 and Supplementary Table S1).

**Fig. 4:**
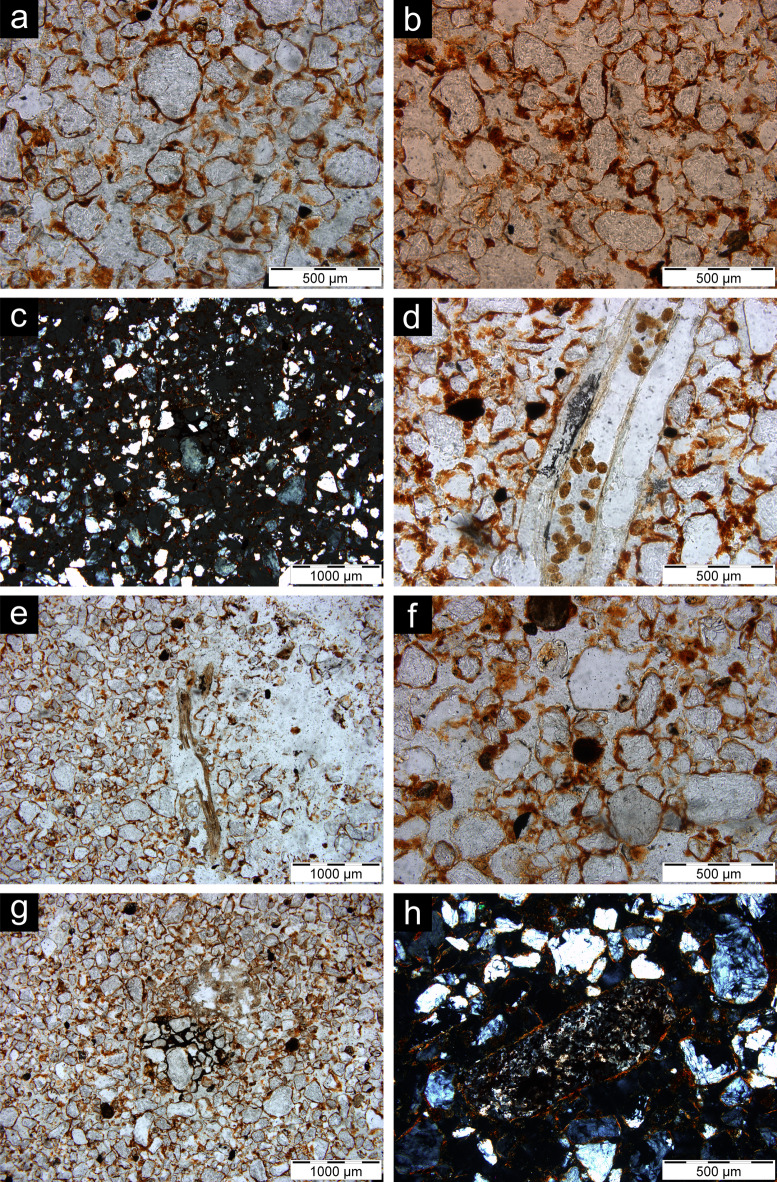
Photomicrographs of representative thin sections from Baden-Baden 2; all images are in plane polarised light, except for **c**) and **h**) that are in cross polarised light. (**a**) Chito-gefuric c/f related distribution from sample 3; (**b**) Intergrain microaggregate microstructure from sample 3; (**c**) Grano- and poro-striated b-fabric from sample 4; (**d**) Excrements of Oribatid mites from sample 1; (**e**) Channel with plant remains from sample 2; (**f**) Nodule of Fe-Mn hydroxide from sample 3; (**g**) Hypo-coating of Fe-Mn hydroxide from sample 4; (**h**) Fragment of hornfels from sample 4.

Micromorphological analysis provided insights into formation and post depositional processes. The micromorphological samples were grouped under three microfacies types (MFT): bioturbated sand, cross-bedded sand, and modern sheetwash (Supplementary Table S2). The bioturbated sand MFT shows abundant sand-sized quartz grains linked and surrounded by reddish clay (*chito-gefuric coarse/fine related distribution*) (Fig. 4a), which in places forms small aggregates in the interstitial spaces between the coarser grains (*enaulic c/f related distribution*). Coarse sand-sized and silt-sized grains of quartz, feldspar and magnetite also occur frequently, making the coarse fraction moderately sorted. Fragments of hornfels from knapping debitage were also observed. Porosity is represented by voids resulting from the loose packing of coarse components and fine fraction (*complex packing voids*) and by abundant cylindrical elongated voids (*channels*), smooth walled pores (*chambers*), and elongated flat voids (*planes*). In between the packing voids, red clay forms micro-aggregates (*intergrain microaggregate microstructure*) (Fig. 4b). The fine fraction is characterised by clay minerals organised in small oriented domains, around grains and pores, or along random straight lines, as evidenced by their birefringence fabric (*stipple speckled*,* grano-striated* and *poro-striated*, and *striated b-fabric*, respectively) (Fig. 4c). Post-depositional processes (*pedofeatures*) are represented mainly by Oribatid mites excrements (Fig. 4d) and plant remains inside channels and chambers (Fig. 4e). Pedofeatures also include Fe-Mn hydroxide features, such as coatings of voids, halos around pores and grains (*hypo-coatings*), and nodules (Fig. 4f-g).

**Fig. 5:**
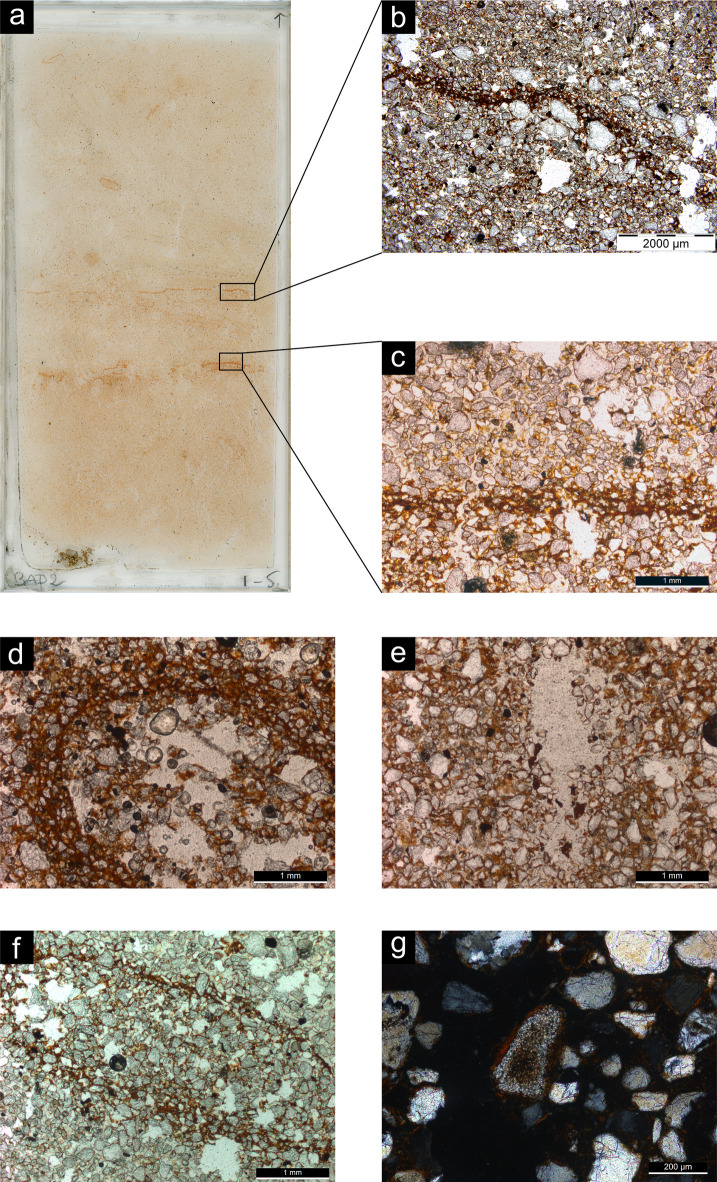
Photomicrographs of representative thin sections from Baden-Baden 2; all images are in plane polarised light, except for **g**), which is in cross polarised light. **a**) Scan of sample 5; **b**-**c**) Horizontal laminae rich in clay; **d**) Hypo-coating of clay from sample 7; **e**) Fragments of clay in a void from sample 10; **f**) Sand infilling from sample 5; **g**) Fragment of hornfels from sample 10.

The cross-bedded sand MFT shares the same overall features as the previous one except for the presence of cross bedding of sand, observed based on the horizontal fabric of the coarse fraction (especially magnetite grains). In addition, micromorphology sample 5 is characterised by the occurrence of thin horizontal beds of clay (*laminae*) (Fig. 5a-c). Pedofeatures include fragments of transported clay (Fig. 5d-e).

**Fig. 6:**
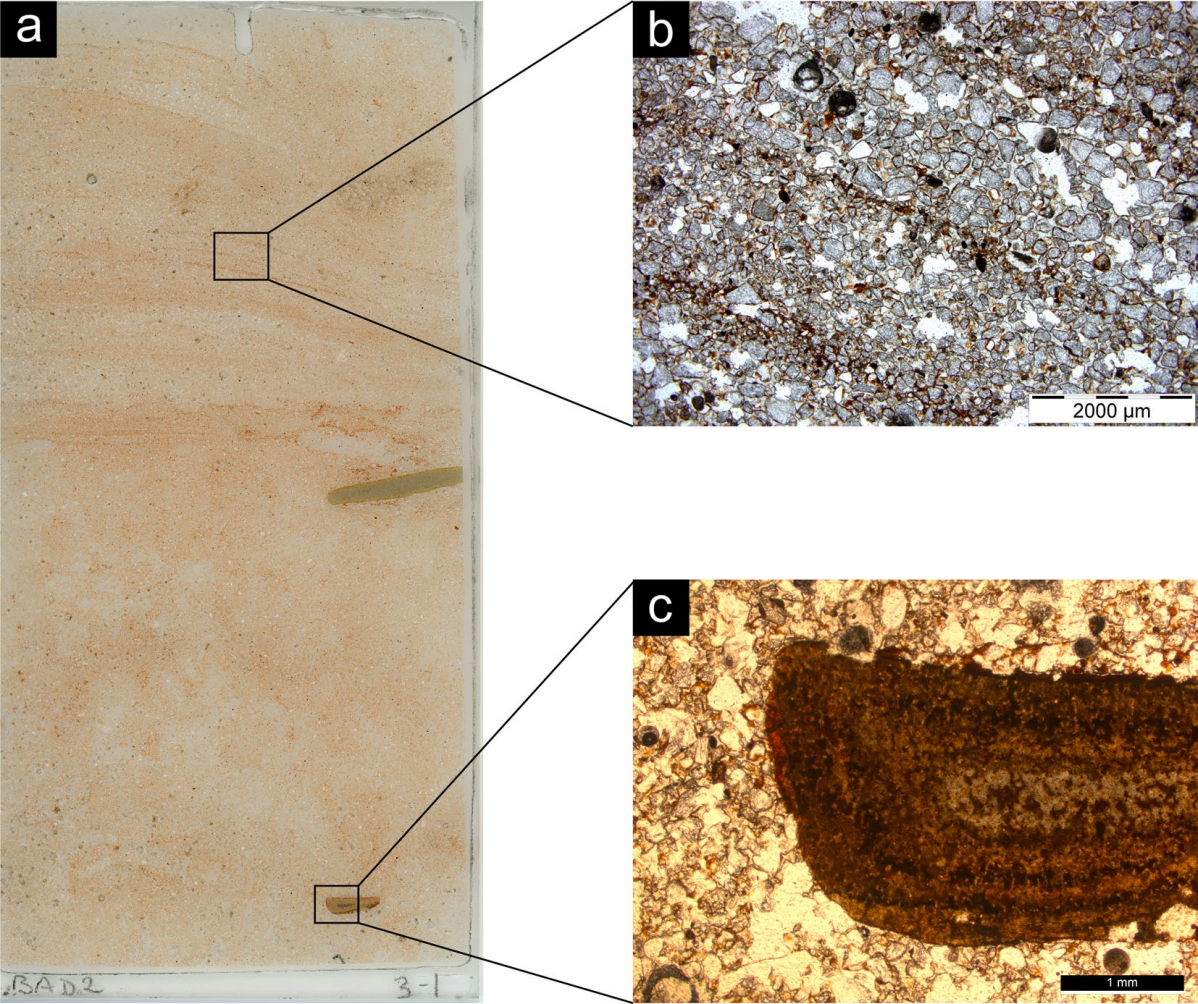
Photomicrographs of representative thin sections from Baden-Baden 2; all images are in plane polarised light. (**a**) Scan of sample 3 − 1, modern sheetwash; (**b**) Laminae rich in clay; (**c**) Reddish patina covering a fragment of hornfels.

The modern sheetwash MFT, represented by sample 3 − 1 from Unit 3 where artefacts in secondary deposition were recovered, differs from the previous two based on the observed sedimentary structures. These feature a large number of thin horizontal beds of sand that alternate with thin laminae of clay (Fig. 6). Pedofeatures are limited to clay coatings and Fe-Mn nodules.

Magnetic susceptibility (MS) measurements allowed to identify breaks in the stratigraphic sequence. MS values from Unit 1 show significant shifts at the contact between orange and red sand, and between Levels 5 and 6 (Fig. 3 and Supplementary Table S3). The latter is invisible to the naked eye. The anhysteretic remnant magnetisation (ARM) confirms the MS results and thus indicates a homogeneous distribution of magnetite grains, except for a large shift between Levels 5 and 6 (Fig. 3).

### Chronology

The OSL dating results are presented in Table 1. They range from 106,100 ± 7,900 years (BAD8, Unit 5-Level 6) to 120 ± 30 years (modern sample, Unit 6-Level 1). Considering the degree of bioturbation indicated by the number of pedofeatures and single grain luminescence analyses, the Finite Mixture Model (FMM)^41^ was applied for equivalent dose (D_e_) calculation, except for the modern sample for which the Central Age Model (CAM)^50^ was applied. For the modern sample, the D_e_ were difficult to measure due to the dim signal, as expected from such young sample, hence the low yield of values that were accepted following our selection criteria (3.5%, Supplementary Table S4). The statistics regarding the single grain selection criteria are available in Supplementary Table S4, as well as the dose recovery (DRT) tests results. The DRT values range from 0.85 ± 17% (BAD4) to 1.08 ± 18% (BAD3 and 7), giving a mean value of 1.03. The OD values obtained on the dose recovery ratios range from 4% (BAD-MOD) to 24% (BAD5) (Supplementary Table S4).

**Fig. 7:**
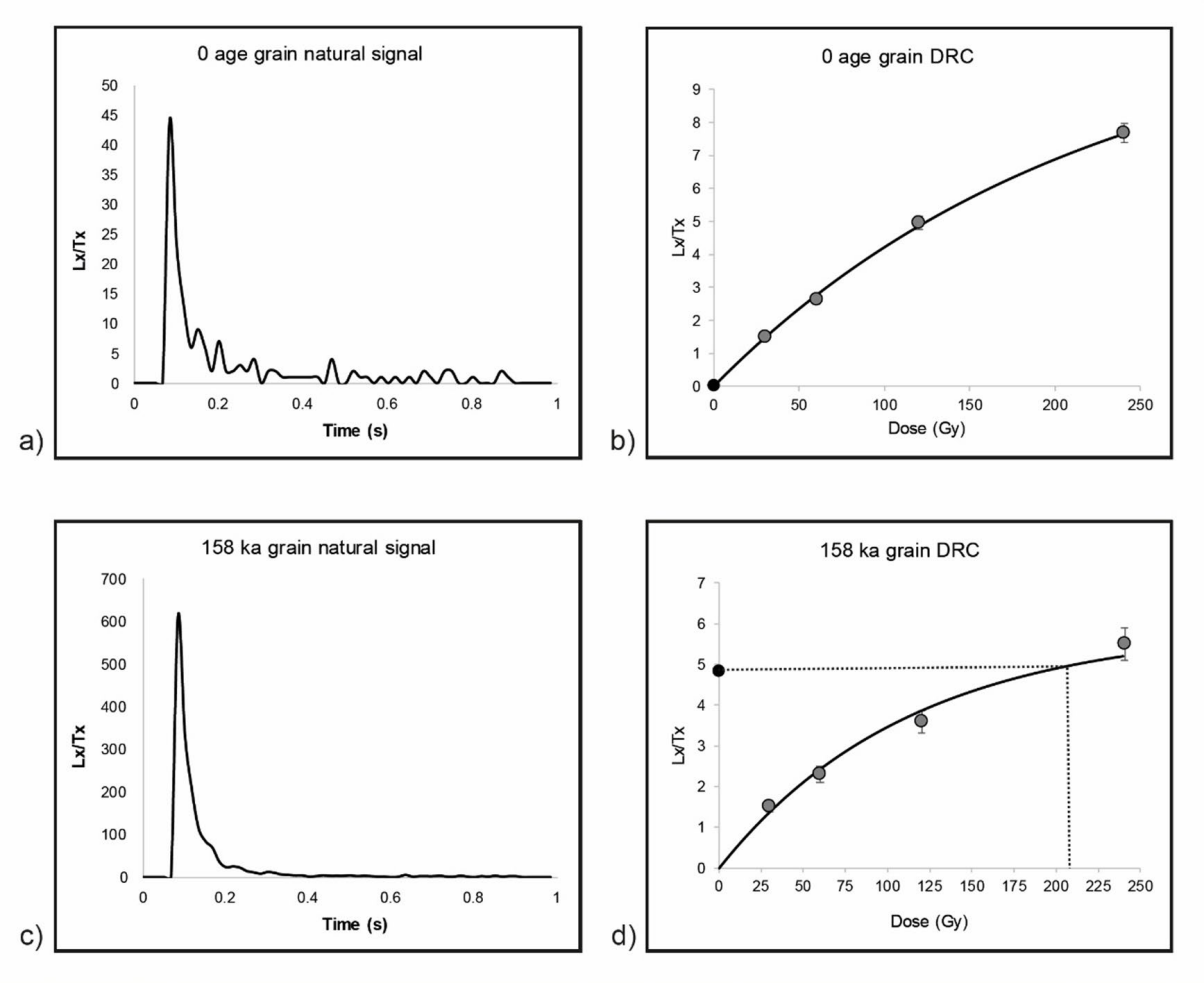
Natural signal (**a**, **c**) and DRC (**b**, **d**) of a zero-age grain (D_e_ = 0.3 ± 0.5 Gy) and of the oldest grain (D_e_ = 191.6 ± 41.3 Gy) from sample BAD1.

Regarding the D_e_, the OD values range from 97% to 131% (Table 1), confirming the extensive mixing of the grains, mainly due to termite activity observed during the excavation (Supplementary Figure S11 and Video 1). In addition, zero-age grains and/or very young grains (D_e_ < 5 Gy) were observed in all samples (Fig. 7). For instance, in the younger sample BAD1, the ages of individual grains range from 0 ka to 158 ka (Fig. 7), and in the oldest sample BAD8, they range from 1.4 ka to 265 ka. The distribution of D_e_ values for each sample is provided in Supplementary Figure S12, and shows that in each archaeological sample, mainly young grains were incorporated into older levels in the section, through the channels dug by termites, and that some older or incompletely bleached grains may also be present. This is supported by the single grain analyses conducted on the modern sample (BAD-MOD). They showed little contamination from old grains (D_e_ < 1.5 Gy, Supplementary Figure S12), suggesting that the channels produced by the termites, associated with the action of the gravity and water circulation, favoured the movement of grains from the top to the bottom of the sequence. In particular, we can observe in all archaeological samples that many grains have a D_e_ < 30 Gy (giving an age range ≤ 28 − 25 ka depending on the sample). Given the type of lithic industry (see lithic artefacts results section), we can exclude the opposite case according to which mainly older grains were incorporated into young levels.

**Fig. 8:**
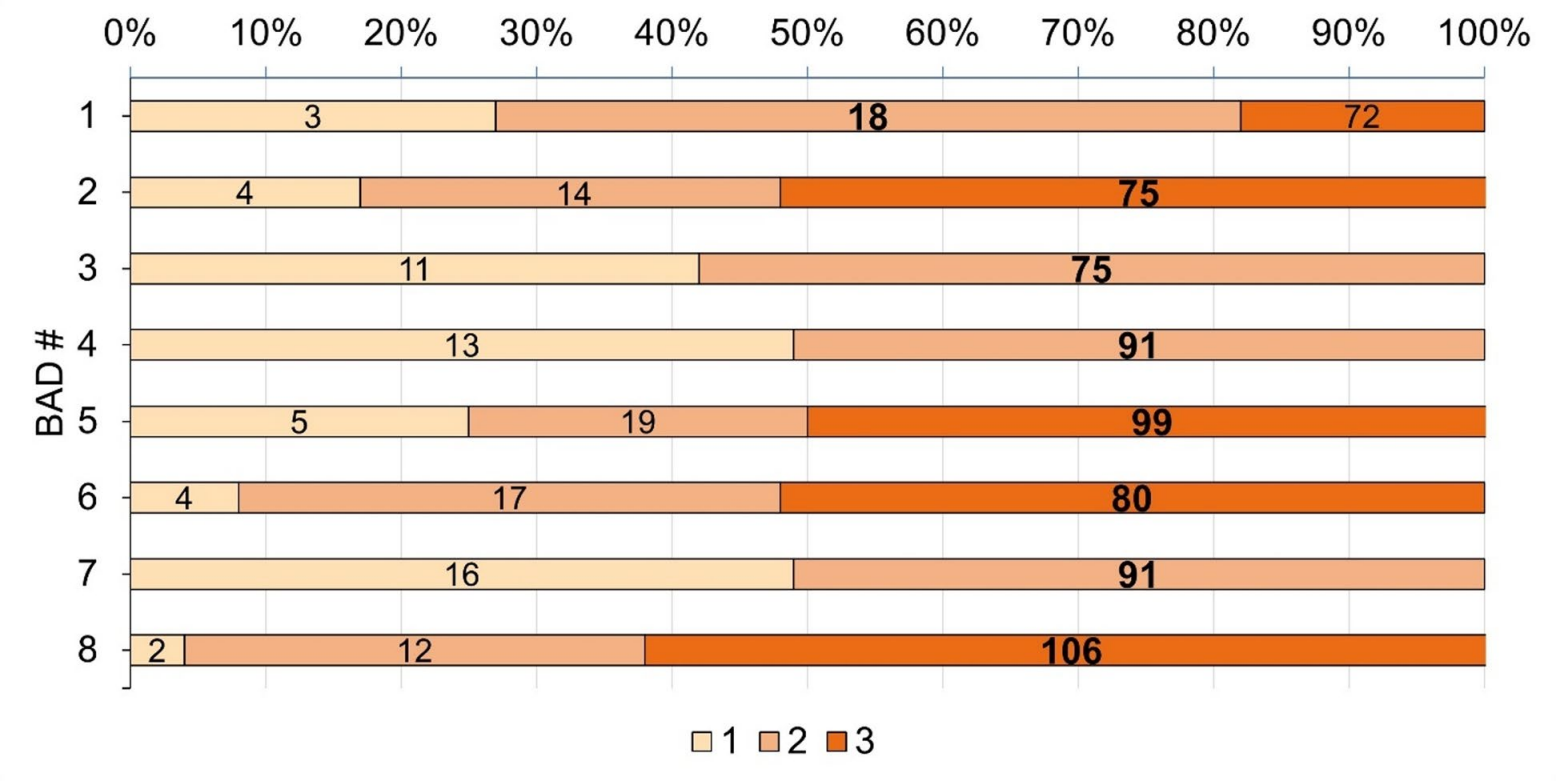
FMM components (from one to three) obtained for the archaeological sediment samples. The bold values are the ages calculated using the main component obtained given by the lowest Bayes Information Criterion (BIC) score^42^.

The FMM was thus applied to extract the main dose component, which appears to be the older one, except for BAD1, and represents between 51% and 63% of the grains (Fig. 8), which provides ages that are in agreement between the two different trenches (Fig. 3). The results of the FMM, including the σ_b_ values used, the different dose components and relative proportion are detailed in Supplementary Table S5. Our results suggest that human occupation at the site started in Level 5 (Unit 1) and Level 7 (Units 5 and 6) at ~ 91 ka, and ended in Level 4 in all units at ~ 75 ka.

**Table 1:** Single grain OSL ages (1 σ). The equivalent doses (D_e_) were measured using the standard 150 μm holes-disc (accepted (*n*) and measured (N) grains). Alpha and beta dose rates were calculated from the radioactive elements measured by gamma spectrometry (U, Th, and K contents, see Table 2) using the conversion factors of Guérin et al.^51^. A water content of 6% was assumed based on the measured values provided in Table 2. Cosmic dose rates were estimated from current burial depths below surface using the equation from Prescott and Hutton^52^. The ages were calculated taking into account beta absorption factors of 0.100 (U), 0.142 (Th), and 0.041 (K)^53^; alpha attenuation factors of 0.14 (U) and 0.17 (Th)^54^; an s_α_-value of 4.5 ± 20%^55^. The Finite Mixture Model (FMM)^34^ was used for all samples (see details in Supplementary Table S5) except for the modern one for which the Central Age Model (CAM)^50^ was applied. BAD # = Baden-Baden 2 sample number. OD = overdispersion. *Minimum age estimate.

BAD #	Depth (cm)	OD (%)	*n*/N	D_e_ (Gy)	Dose rate (µGy/a)					Age (a)
					Alpha	Beta	Gamma	Cosmic	Total	
1	50	121 ± 9	102/1000	21.9 ± 1.8	22 ± 3	621 ± 8	371 ± 9	200 ± 10	1214 ± 13	18.000 ± 1.700*
2	70	121 ± 9	95/1000	90.3 ± 5.2	23 ± 3	621 ± 7	364 ± 9	191 ± 10	1199 ± 12	75.300 ± 5.500
3	80	109 ± 8	107/1000	88.1 ± 6.7	22 ± 3	601 ± 6	366 ± 9	188 ± 10	1177 ± 11	74.800 ± 6.500
4	90	110 ± 8	104/1000	108.5 ± 7.9	23 ± 3	613 ± 7	376 ± 9	185 ± 10	1197 ± 12	90.600 ± 7.700
5	115	131 ± 11	78/1000	117.3 ± 9.1	22 ± 3	614 ± 6	369 ± 9	179 ± 10	1184 ± 11	99.000 ± 8.800
6	80	104 ± 7	106/1000	89.4 ± 5.9	21 ± 3	608 ± 6	297 ± 7	188 ± 10	1114 ± 10	80.200 ± 6.300
7	95	97 ± 7	97/1000	103.1 ± 7.1	22 ± 3	625 ± 5	305 ± 7	184 ± 10	1136 ± 9	90.800 ± 7.400
8	120	124 ± 9	90/1000	115.8 ± 7.0	20 ± 3	588 ± 5	306 ± 7	178 ± 10	1091 ± 9	106.100 ± 7.900
MODERN	10	98 ± 24	35/1000	0.14 ± 0.04	21 ± 3	757 ± 7	163 ± 4	258 ± 10	1199 ± 9	120 ± 30

The U, Th, and K contents measured in the sediment samples and used to derive the alpha and beta dose rates, as well as the measured water content, are presented in Table 2. As expected for a sand dune, no significant variation in radioelement content is observed^25^, which indicates that the dosimetry in this environment is homogeneous. This is also supported by the in-situ gamma dose rate values (Table 1), which range from 297 ± 7 to 376 ± 9 µGy.a^− 1^. Only the modern sample has a significantly lower dose rate, of 163 ± 4 µGy.a^− 1^, which can be explained by the position of this sample, collected 10 cm below the current surface. Considering that the penetration power of gamma rays in a sedimentary environment is ca. 30 cm, the gamma dose rate is necessarily lower for the modern sample.

**Table 2:** U, Th, and K contents of sediment samples analysed with a laboratory gamma-ray spectrometer equipped with a high-resolution, broad energy Ge (BEGe) detector, and measured water content (water/dry weight of sediment %).

BAD #	U (ppm)	Th (ppm)	K (%)	Water content (%)
1	1.06 ± 0.02	3.56 ± 0.05	0.58 ± 0.01	7
2	1.10 ± 0.01	3.50 ± 0.04	0.58 ± 0.01	6
3	1.08 ± 0.01	3.49 ± 0.04	0.55 ± 0.01	6
4	1.08 ± 0.01	3.57 ± 0.04	0.57 ± 0.01	6
5	1.05 ± 0.01	3.52 ± 0.04	0.57 ± 0.01	7
6	0.89 ± 0.01	3.65 ± 0.04	0.59 ± 0.01	5
7	0.92 ± 0.01	3.75 ± 0.03	0.60 ± 0.01	3
8	0.82 ± 0.01	3.53 ± 0.03	0.58 ± 0.01	4
MODERN	0.89 ± 0.01	3.52 ± 0.04	0.80 ± 0.01	4

### Plant biomarkers

**Fig. 9:**
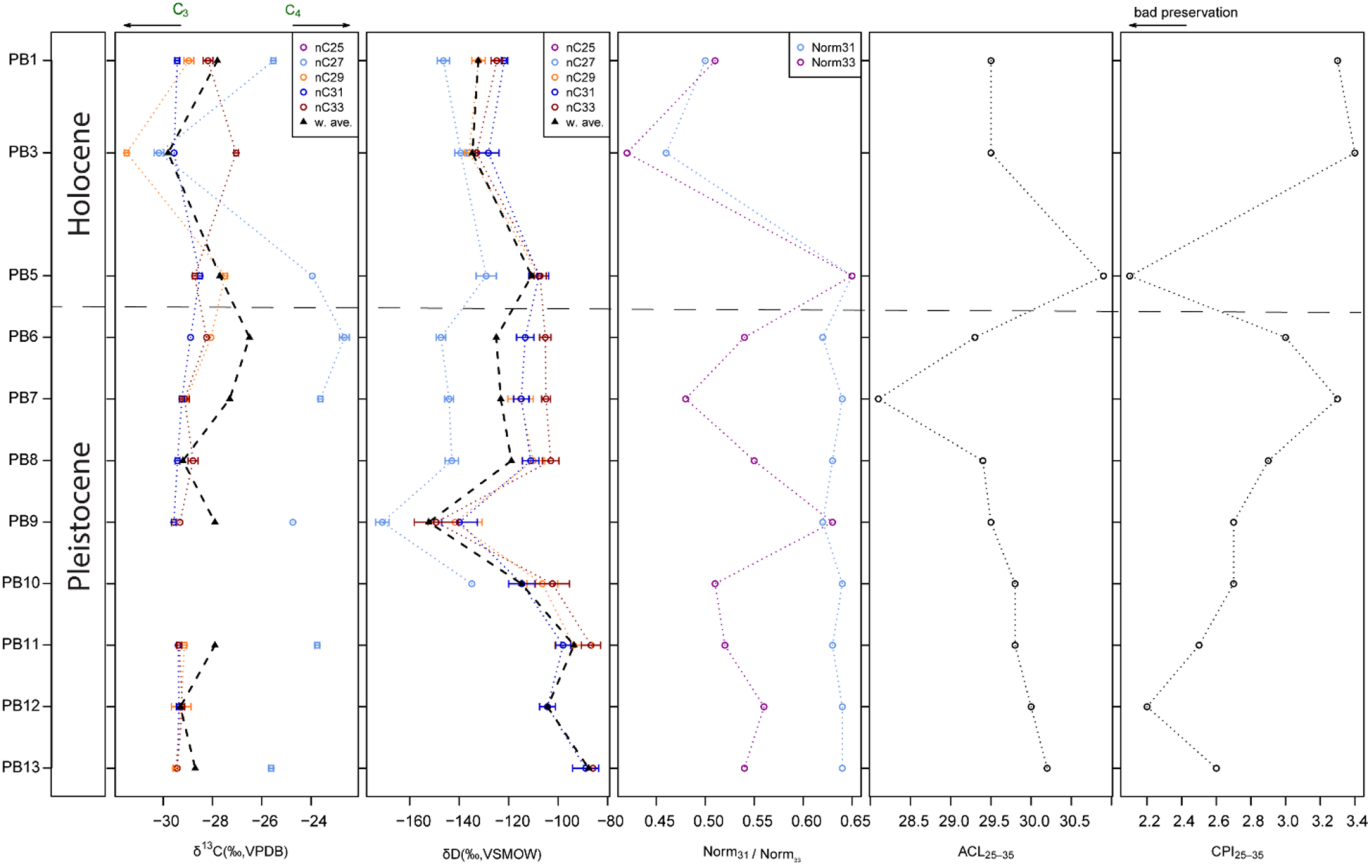
Leaf wax *n*-alkane results for Baden-Baden 2. From left to right: sample name; δ^13^C isotope values and their weighted average; δD isotope values and their weighted average; Norm_31_ and Norm_33_ index; Average Chain Length (ACL) of the C_25_-C_35_ *n*-alkanes; Carbon Preference Index (CPI) of the C_25_-C_35_ *n*-alkanes.

The concentration of biomarkers in the samples was relatively low, ranging from 380.9 ng to 964.9 ng per gram of sediment (Dataset 1). The plant wax biomarker distributions show an odd-over-even ratio with a clear odd dominance. Higher chain lengths (*n*-C_27_ to *n*-C_33_) dominate in all but one sample, indicating a mainly terrestrial plant wax source. Average chain length (ACL_25 − 35_) ranges from 28.1 to 30.9 (average 29.6 ± 0.6, *n* = 11) (Table 3; Fig. 9). The large variation in ACL is also reflected in the dominant homologue, which varies by sample. While in the older samples the *n*-C_31_ homologue dominates (PB 10-PB 13), in the younger samples *n*-C_27_ has the highest abundance. Exceptions are PB 7, where *n*-C_25_ is dominating, and PB 5, where *n*-C_33_ is the most abundant homologue. The carbon preference index (CPI) of the C_25_ − C_35_ *n*-alkanes ranges between 2.1 and 3.4 (2.8 ± 0.4, *n* = 11) (Table 3). As all CPI values are < 2, this indicates good preservation and little degradation of the terrestrial *n*-alkane chains. The Norm_31_ (average 0.6 ± 0.1) and Norm_33_ (average 0.5 ± 0.1) indices do not show much variation between samples (Table 3; Fig. 9).

The carbon and hydrogen stable isotope results for *n*-C_27_, *n*-C_29_, *n*-C_31_, and *n*-C_33_ are given in Table 3, and the full results can be found in Dataset 1. Due to the low concentration of *n*-alkanes in the samples, not every homologue could be measured for carbon and hydrogen, and in several duplicates. For these samples, priority was given to measure hydrogen isotope values. The δ^13^C results exhibit little variation throughout the sequence (Fig. 9) with the C_27_ *n*-alkanes displaying the highest δ^13^C values in all but one sample (sample PB 3). The δ^13^C values range from – 22.7‰ to – 30.2‰ in C_27_ and from – 27.5‰ to – 31.5‰ in C_29_, but range only 1.1‰ (– 28.5‰ to – 29.6‰) in C_31_ and 2.4‰ (– 27.0‰ to – 29.4‰) in C_33_. In the hydrogen isotope values, *n*-C_27_ is consistently showing the lowest δD values. The δD values range from – 129.1‰ to – 171.1‰ in C_27_, and from – 93.8‰ to – 141.8‰ in C_29_, as well as from – 88.9‰ to – 139.9‰ in C_31_, and from – 86.0‰ to – 149.4‰ in C_29_. On average, that is 51.1‰ variation within the results of a homologue.

**Table 3:** Indices and stable isotopes results for biomarkers from Baden-Baden 2. From left to right: Sample name and depth, Sum (ng/µl), Norm_31_ and Norm_33_ indices; Average chain length (ACL) of the C_25−35_ *n*-alkanes; Carbon Preference Index (CPI) of the C_25_-_35_ *n*-alkanes; stable isotope results for C_27_ to C_33_. All isotope results are given as the average value of all measurements per sample in per mill (‰), with the δ^13^C values given against VPBD, δD values are given against VSMOW. The standard derivation (STDEV) is provided and the number of measurements is given in brackets.

Sample	Age	Sum (ng/µl)	Norm_31_	Norm_33_	ACL_25−35_	CPI_25−35_	δ^13^C nC_27_	δ^13^C nC_29_	δ^13^C nC_31_	δ^13^C nC_33_	δD nC_27_	δD nC_29_	δD nC_31_	δD nC_33_
PB1	120 a	120.8	0.50	0.51	19.5	3.3	-25.53 ± 0.1‰ (3)	-28.96 ± 0.2‰ (3)	-29.43 ± 0.1‰ (3)	-28.18 ± 0.2‰ (3)	-146.4 ± 2.5‰ (3)	-132.2 ± 2.7‰ (3)	-121.7 ± 1.3‰ (3)	-124.8 ± 2.3‰ (3)
PB3	Holocene	105.7	0.46	0.42	29.5	3.4	-30.17 ± 0.2‰ (3)	-31.48 ± 0.1‰ (3)	-29.56 ± 0.0‰ (3)	-27.04 ± 0.1‰ (3)	-139.4 ± 2.4‰ (3)	-136.0 ± 1.8‰ (3)	-128.2 ± 4.3‰ (3)	-132.9 ± 0.4‰ (3)
PB5	Holocene	154.7	0.65	0.65	30.9	2.1	-23.95 ± 0.0‰ (3)	-27.49 ± 0.1‰ (3)	-28.51 ± 0.1‰ (3)	-28.72 ± 0.1‰ (3)	-129.1 ± 4.1‰ (3)	-107.9 ± 3.2‰ (3)	-107.8 ± 4.0‰ (3)	-107.3 ± 2.5‰ (3)
PB6	≥ 18 ± 2 ka	75.9	0.62	0.54	29.3	3.0	-22.65 ± 0.2‰ (2)	-28.06 ± 0.0‰ (2)	-28.89 ± 0.0‰ (2)	-28.24 ± 0.0‰ (2)	-147.4 ± 1.9‰ (3)		-113.3 ± 3.5‰ (3)	-105.2 ± 2.3‰ (3)
PB7	< 80 ± 6 ka > 18 ± 2 ka	640.6	0.64	0.48	28.14	3.3	-23.62 ± 0.1‰ (3)	-29.06 ± 0.1‰ (3)	-29.24 ± 0.1‰ (2)	-29.13 ± 0.2‰ (3)	-144.1 ± 1.8‰ (4)	-115.2 ± 5.1‰ (2)	-114.9 ± 3.1‰ (4)	-104.9 ± 1.8‰ (3)
PB8	< 80 ± 6 ka > 18 ± 2 ka	71.9	0.63	0.55	29.4	2.9			-29.42 ± 0.1‰ (2)	-28.79 ± 0.2‰ (2)	-143.0 ± 2.7‰ (2)	-110.1 ± 4.6‰ (3)	-111.1 ± 3.3‰ (2)	-103.0 ± 3.3‰ (2)
PB9	80 ± 6 ka	104.6	0.62	0.63	29.5	2.7	-24.74 ± 0.0‰ (2)	-29.54 ± 0.1‰ (2)	-29.57 ± 0.1‰ (2)	-29.32 ± 0.0‰ (2)	-171.1 ± 2.7‰ (4)	-141.7 ± 10.9‰ (4)	-139.9 ± 7.2‰ (4)	-149.3 ± 8.9‰ (3)
PB10	91 ± 7 ka	65.5	0.64	0.51	29.8	2.7					-134.9 (1)	-106.4 ± 6.2‰ (3)	-114.7 ± 5.3‰ (2)	-102.3 ± 6.9‰ (2)
PB11	< 106 ± 8 ka > 91 ± 7 ka	96.8	0.63	0.52	29.8	2.5	-23.75 ± 0.1‰ (3)	-29.14 ± 0.1‰ (3)	-29.39 ± 0.0‰ (3)	-29.35 ± 0.1‰ (3)		-93.8 ± 7.5‰ (4)	-98.0 ± 3.0‰ (4)	-86.7 ± 3.9‰ (3)
PB12	106 ± 8 ka	61.1	0.64	0.56	30.0	2.2		-29.27 ± 0.4‰ (2)	-29.36 ± 0.1‰ (2)	-29.22 ± 0.1‰ (2)			-104.3 ± 3.2‰ (4)	
PB13	> 106 ± 8 ka	76.2	0.64	0.54	30.2	2.6	-25.62 ± 0.1‰ (2)	-29.50 ± 0.1‰ (2)	-29.43 ± 0.0‰ (2)	-29.43 ± 0.0‰ (2)			-88.9 ± 5.3‰ (4)	-85.9 (1)

### Lithic artefacts

The collection from Baden-Baden 2 is composed of 1153 lithic artefacts. Of these, 560 originate from a controlled stratigraphic context, while the remainder represent a surface collection from the eroded area to the south of the excavation units. In the sample of 467 artefacts examined in this study (Table 4), hornfels is the predominant raw material (*n* = 444), followed by dolerite (*n* = 13), syenite (*n* = 5), quartz (*n* = 2), chert (*n* = 1), quartzite (*n* = 1), and banded ironstone (*n* = 1) (Supplementary Table S6). All of the hornfels artefacts display a red patina that is often weathered at the edges, revealing a yellow-greenish patina underneath.

**Fig. 10:**
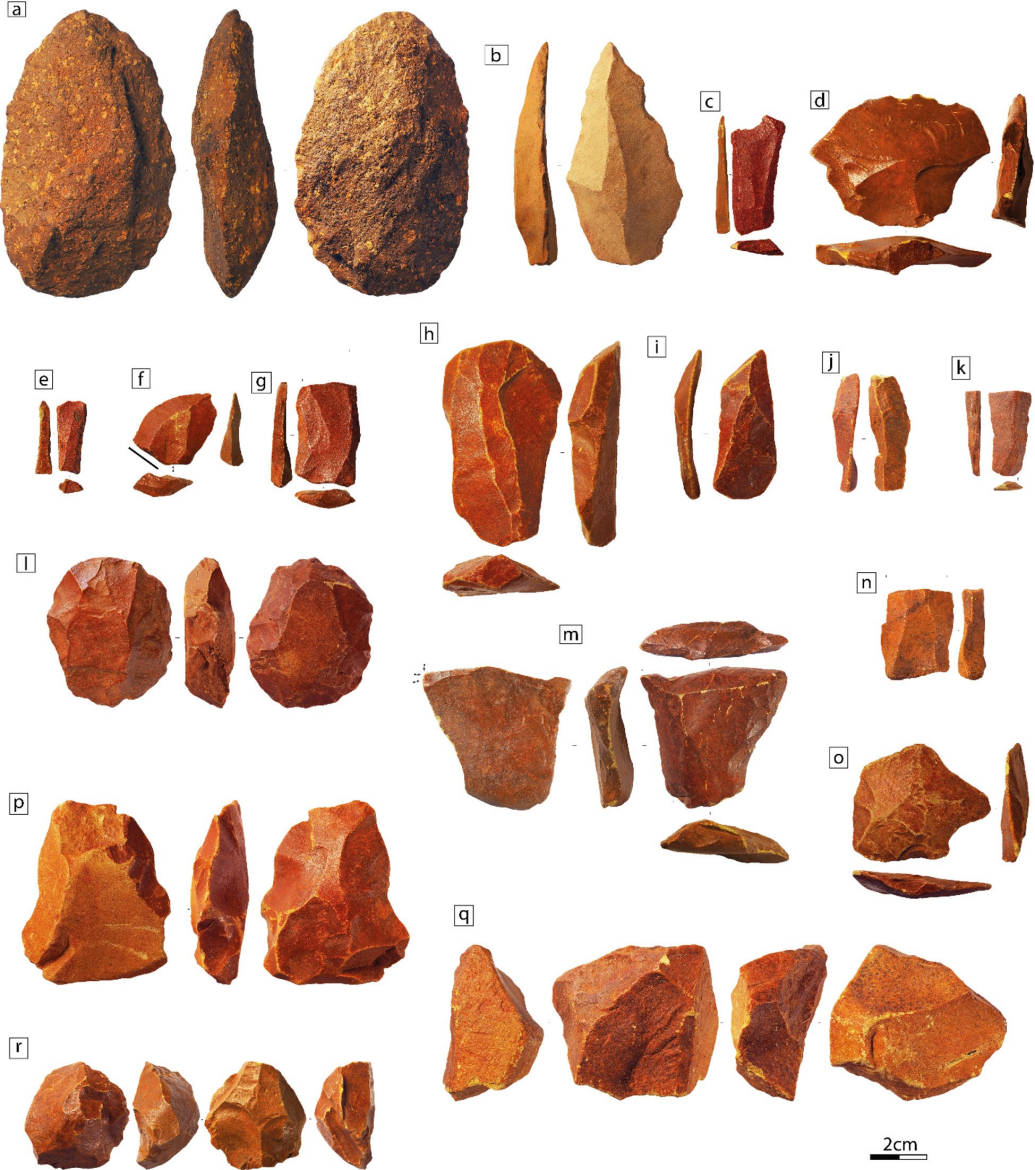
Examples of lithic artefacts from Baden-Baden 2. **a**, **b**: surface collection; **c** to **f**: L3; **g**, **h**: L4; **i** to **m**: L5; **n** to **q**: L6; **r**: L7.

The surface finds include artefacts that can be assigned to the ESA and MSA. Notably, these include a small handaxe (Fig. 10a) and a polyhedron made of syenite, several Levallois cores and Levallois-like blanks, and one triangular element (Fig. 10b). The artefacts from controlled stratigraphic contexts exhibit comparable features, indicating the presence of MSA components within the assemblage^56–59^. The study of materials from the various levels reveals a general tendency towards the production of elongated blanks from prepared cores (Fig. 10e, g-k, r), and a low frequency of edge modifications (*n* = 11; 2.3%) (Fig. 10k). Platforms frequently exhibit preparations, dihedral or faceting (19%, 13%), although they are not systematically prepared, as plain butts are also observed (18%) (Supplementary Table S7). Blanks are largely obtained through unipolar and unipolar convergent reduction strategies (16% to 58%) (Fig. 10h-i) (Supplementary Table S8). Bipolar modalities are also observed (10% to 25%) (Fig. 10c). Specifically, in Level 6 from the 2024 excavation, they are more frequently represented than unipolar and unipolar convergent modalities (18%, 12%, and 16%, respectively). This occurrence implies a predetermined and predetermining character of the elongated blanks, and also suggests the presence of cores structured by two opposing striking platforms. These settings directly influence the overall morphology of the blanks (e.g., elongated blanks), resulting in a rectilinear profile, parallel edges, and often a trapezoidal cross-section (Fig. 10c, e, g, h). The management of distal convexities is therefore well controlled, indicating that the morphology of these elements is a planned feature. In addition, bladelet production (width < 15 mm) seems to occur in a few levels (e.g., Level 5 and 6 from 2024, and Level 3 and 4 from 2023). These elements are represented both by blanks and cores (Fig. 10e, j-k, m, r), which appear to be obtained from prepared cores but also from burins (*n* = 2) (Fig. 10m). Flake production is another notable feature of the assemblage (Fig. 10d, f, o). These elements are mostly produced from parallel and inclined cores^60^ (Fig. 10f, p-q). Levallois methods are present in Level 5 and 6 from 2024 and Level 3 and 4 from 2023 (Fig. 10d, l, o), where they were observed mainly on blanks (*n* = 8) but also cores (*n* = 2). Typo-Levallois blanks and cores are also noted^61^ (Fig. 10q). The representation of the different phases of these reduction sequences varies across levels. However, despite the limited number of artefacts in this assemblage, cortical, shaping, and maintenance blanks are occasionally represented (Table 4), indicating that part of the exploitation phases of the core reduction took place within the excavated area (i.e. elongated blanks and flake production).

**Table 4:** Breakdown of selected lithic artefacts by Unit (U) and Level (L) at Baden-Baden 2. Units 1-2-3 were excavated in 2023; Units 5–12 were excavated in 2024. Note that L4 and L5 in Units 1–2 do not correspond to the same Levels in Units 5–12 due to the slope of the dune. Artefacts in U3, L2 were found in secondary deposition.

Flake	U5-12, L4	U5-12, L5	U5-12, L6	U5-12, L7	U1-2, L3	U1-2, L4	U1-2, L5	U3, L2	Surface	Total
	8	6	10	1	2	3	4	1	23	58
Levallois		1	4		1	1	1		6	14
Kombewa	2	1	2					1		6
Fragmented flake	4	2	3		2	4		5	7	27
Blade/bladelet	4	12	7	2	4	5		29	37	100
Triangular									1	1
Elongated blanks	2	4	8	1		1	1	8	13	38
Crested blanks	2		2			1		3	4	12
*Débordant*	4	3	7	2		4		6	36	62
Pseudo P.-Levallois	1		5			1		5	4	16
Backed blank		2						2	27	31
Natural back									5	5
Cortical	3	2	3		1			1	3	13
Core	1	3	4	1	2	2		4	65	82
Indeterminate									2	2
Total	31	36	55	7	12	22	6	65	233	467

## Discussion

*Formation processes*. The geoarchaeological and chronological analyses conducted on the sediment samples collected at Baden-Baden 2 allowed the reconstruction of its formation and post-depositional processes over time. FTIR spectroscopy, image analysis, and micromorphology revealed that the large amount of quartz sand exhibiting a relatively high degree of roundness, and spatially organised in cross beds throughout the sequence, was accumulated by aeolian deposition^27,62^, consistent with the geomorphological layout of the area. Much of the cross bedding was disrupted by post-depositional bioturbation of the sediments caused by root channels and termite activity, represented by channel and chamber voids. In addition, the porosity created by such disturbances promoted the translocation of the fine and coarse fractions and the formation of hydroxides through groundwater movement, indicative of periodically moist conditions^63^.

*Chronology*. With regard to the chronological results, the use of the FMM allowed to identify the number of dose components, included between 2 and 3 depending on the samples (Fig. 8 and Supplementary Table S5), and highlighted that the main dose component is always the oldest one except for the younger archaeological sample BAD1, for which an age of 18 ± 2 ka was obtained using the main component (Table 1). For this specific sample, the oldest component gives an age of 72 ± 11 ka but is only based on 18% of the grains (Fig. 8). Both ages are consistent with the context, however, as a precaution, we may consider the age of 18 ka, which represents 55% of the grains, as a minimum estimate for the top of the red sands. The other ages, whose older components constitute 51% to 63% of the grains, are in stratigraphic order and range from 106 ± 8 ka (Level 8, Units 5 and 6) to 75 ± 6 ka (top of Level 4, Units 1 and 2) (Fig. 3).

According to these results, each dated level was mainly contaminated by younger grains, consistent with field and micromorphology observations regarding the movement of grains from the top to the bottom of the sequence through the channels dug by termites and plants. Considering that the ages are in stratigraphic order and in agreement between the 2023 and 2024 trenches, we can conclude that the approach used to determine the σ_b_ was appropriate.

Furthermore, the ages calculated using the main component of the lowest BIC score with a fixed σ_b_ = 0.20 (see details in Supplementary Table S9) show some stratigraphic inversion (circles in Supplementary Figure S13); some of the ages are overestimated using the oldest components and a fixed σ_b_ = 0.20 for BAD2 to 8 (squares in Supplementary Figure S13). However, using the value of σ_b_ that gives the lowest BIC score and the main component, the ages are in stratigraphic order and in agreement between the two trenches (diamonds in Supplementary Figure S13; Fig. 3). These observations support the use of a range of σ_b_ to determine the optimal combination of σ_b_ and *k*.

Another potential issue must be addressed about the dose rate. Guérin et al.^64^ expressed concerns regarding the dose rate used to calculate the age of each component, i.e., the dose rate measured today in the dated level may not be representative of the one received by the grains prior to mixing. Nevertheless, in the case of Baden-Baden 2, the dose rate is homogeneous within the sequence, showing little variations in both U, Th, and K contents used to derive the beta dose rate (Table 2) and in the gamma dose rate measured *in situ* (Table 1). The total dose rates range from 1214 ± 13 µGy.a^− 1^ (BAD1) to 1091 ± 9 µGy.a^− 1^ (BAD8), likely having little impact on the age calculation. For example, the age of the youngest component (D_e_ = 2.60 ± 0.89 Gy, proportion = 4%, Supplementary Table S5) of the oldest sample (BAD8) is 2.4 ± 0.8 ka using the dose rate of Level 8 where BAD8 was collected (Fig. 8), and 2.2 ± 0.7 ka using the one from Level 1, where the modern sample was collected. This is not surprising, considering that the sequence consists of an accumulation of red sand presumably from the Kalahari Basin, where no erosional unconformities could be discerned based on the sedimentology, and for which the FTIR spectra showed that it is mainly composed of quartz, followed by clay minerals (Supplementary Figure S8). Furthermore, the high degree of roundness evidenced by the HSC values is consistent with long distance transport by wind and may suggest an optimal bleaching of the quartz grains.

The results of OSL dating showed that the stratigraphic sequence excavated in Units 1–2 and 5–12 is ~ 106,000 years old. An earlier dating programme showed that the foot of the dune, near the present-day spring, is ~ 164,000 years old at ~ 5 m depth^35^, based on which it can be assumed that aeolian deposition at the site dates back to at least the late Middle Pleistocene, as observed also at Florisbad, located in a similar dune setting^17,18^. Human occupation at Baden-Baden 2 started at ~ 91 ka (Level 5 in Unit 1 and Level 7 in Units 5–6, ~ 95–110 cm depth), based on OSL samples BAD4, BAD5, and BAD7 (Fig. 3). These samples overlap with the purported occupation surface displayed in Supplementary Figure S6, on which artefacts were found lying flat in clusters. Micromorphology results lend support to this interpretation based on sample 5, which features at least two separate events of sheetwash at the same depth of the clusters of artefacts (Fig. 5a-c). These accumulations of clay laminae are the result of sediment transport by surface waters during heavy rainfall on exposed substrates^65,66^, which can be interpreted as occupation surfaces based on the occurrence of clusters of artefacts. Similar examples of clay laminae can be observed in micromorphology sample 3 − 1 from Unit 3 (Fig. 6), which is the result of several cycles of sheetwash and offers a modern parallel for the sedimentary structure, and in modern and Pleistocene samples documented at Florisbad^17^. A shift in MS between Levels 5 and 6 in Unit 1 is in agreement with the micromorphology results. The last occupation is dated to ~ 75 ka (BAD2; Fig. 3), thus at the end of MIS 5^5^. The results of MS/ARM and micromorphology indicate that the boundary between orange sand and red sand, likely younger than ~ 18 ka (BAD1; Fig. 3), was caused by a change in the source of sediment, exemplified by the different colours and magnetic properties of the fine fraction. A similar abrupt turnover of sediments was observed in the terminal Pleistocene sequence at Florisbad^17^ and may be related to the Last Glacial Maximum.

*Palaeoenvironments*. The preservation of plant wax biomarkers at Baden-Baden 2 is good, with CPI values consistently higher than 2 (Table 3; Fig. 9) and a clear odd-over-even dominance. Concentrations of *n*-alkanes per sample are low but this is not surprising considering the dominance of sand as the main sediment component^67^. Aeolian deposition is the accumulation agent for the sediments, and we assume that the majority of plant waxes were incorporated into the sediments by a mix of wind transport and through local plant decomposition. Considering that Annaspan is one the largest pans in the Free State, in an area of high density of pans, we can assume that the vegetation signal most likely is influenced by changes in pan vegetation. The site is located on the boundary of the Vaal-Vet sandy grasslands and the western Free State clay grasslands^7^ with *Phragmites australis* in moister parts close to groundwater and dry grass communities, as well as karroid shrubs but very little to no tree cover. Today the site is located in the Summer Rainfall Zone (SRZ) with mean annual precipitation of 380–530 mm with high summer temperatures and frost in winter^35^. We cannot exclude humans altering the signal by transporting plants to the site. However, the human impact at the site seems minimal and was not of a long-term settlement. Today, wind blows mainly from the east, and rainfall originates over the Indian Ocean^68^. This study shows the impact of bioturbation on moving sand grains in the profile. Therefore, we have to consider that some plant wax biomarker molecules were also moved, and for that reason our signal could be a mix of different time periods.

The δ^13^C signal is very stable with little variation before the Holocene. This could indicate overall a relatively similar plant cover during the Pleistocene. The δ^13^C values indicate a mix of C_3_ and C_4_ plants in the environment throughout, but with relatively low δ^13^C values in the range of C_3_ plants for all homologues except C_27_. These values are only slightly higher than for the Pleistocene levels at Ha Makotoko in Lesotho^69^. However, grasses generally produce less *n*-alkanes than other plant types and therefore may be underrepresented in the plant wax biomarker samples. Additionally, CAM plants such as Aizoaceae have δ^13^C values similar to C_3_ plants^70,71^. The offset between C_27_ and the rest of the homologues suggests that these two biomarkers come from multiple biosynthetic sources, potentially reflecting variability in input from C_3_ woody plants and C_3_ and C_4_ graminoids^72,73^.

The δD data for the biomarkers are highly synchronised, except for C_27_, which has consistently lower δD values (although we have no δD data for C_27_ in the lowermost samples due to low concentrations). This suggests that plants had a shared water source and experienced similar environmental conditions with one group with high C_27_ abundance potentially accessing another water source. However, grasses have shorter roots than trees and shrubs which can access deeper water^74^. The δD values are slightly lower in the Holocene layers than in most of the Pleistocene ones, which might suggest slightly wetter conditions during the Holocene. However, the calculated indices, representing aridity/humidity, do not show the same trends. Again, the different influences of plant types must be taken into account, including the greater water-use efficiency of C_4_ plants, which can result in lower δD values, as has been shown in Holocene studies in southern Africa^69,75^.

Overall, the plant biomarker record can be divided into three phases, which match the three phases between changes in sediment supply identified by MS. The lowermost samples PB10-PB13 are dominated by the C_31_ homologue. The Norm_31_, Norm_33_ and ACL_25−35_ indices are very stable during this time, with the values similar to modern sediment samples from dry, grass-dominated regions^76–78^. These samples have the highest δD values of the sequence. Since δD is influenced by a range of factors, this could hint at increased aridity, evaporation or temperatures as well as a strong seasonal summer rainfall regime or a change in plant composition^72,78–80^. In samples PB6 to PB9, the C_27_ homologue is the most abundant. Together with the MS data this could either indicate a change in predominant source of the sediments or a change in climate regime, representing a different time period. This drives the weighted average of the stable isotope values, where two samples stand out. PB9 has the lowest δD values of the sequence, however, the high Norm_33_ value indicates aridity in this sample. The low δD value might reflect high amounts of rainfall. Sample PB7 is the only one dominated by the C_25_ homologue, indicating the input of submerged aquatic plants^81^. The low Norm_33_ and ACL_25−35_ values further support the influence of standing water in this sample. Altogether, the Pleistocene environment at Baden-Baden 2 is very stable, with very few changes in plant composition and grassier, dryer conditions in the older layers.

The Holocene samples PB1, PB3, and PB5 show higher variation in stable isotope values and indices than the Pleistocene samples, and differences again in homologue abundance. Together with MS and micromorphology, this can indicate a change in sediment source. PB5 is dominated by C_31_ and C_33_ homologues, as well as high Norm_31_, Norm_33_, and ACL_25−35_ values, indicating a rather arid, grassy environment. PB5 is in the same layer as BAD1 (Fig. 3), indicating a Late Pleistocene age. In contrast, PB3 has very low Norm_31_ and Norm_33_ as well as some of the lowest δ^13^C values of the sequence. The pattern of C_27_ being substantially higher in δ^13^C than the other homologues, which is consistent in all other samples, is disrupted, even though C_27_ is the most abundant homologue. This indicates vegetation changes or changes in plant biomarker source during the Late Pleistocene or Holocene, however, we do not have age control for the PB3 sample. PB1 is the topmost sample and represents the last 120 years. None of our samples can be securely linked to the pollen study at nearby Baden-Baden by van Aardt et al.^35^, which showed a treeless, grassy karroid environment around 6.2 ka as well as fynbos type pollen present at ~ 25 ka, indicating cooler grassy conditions. In summary, the Holocene plant environment at Baden-Baden 2 is fluctuating, with evidence for influences from different plant wax sources.

*Lithic industry*. The Baden-Baden 2 lithic assemblage is characterised by a strong emphasis on blade/bladelet and flake production. Blanks are often produced from prepared inclined or parallel cores^60^ and Levallois production occurs occasionally. Overall, the assemblage is distinct from post-MIS 5 industries and from the earlier Fauresmith technocomplex (*sensu* Lombard et al.^57^), and shares common features with pre-Still Bay industries (defined here as all the MSA lithic industries earlier than the Still Bay technocomplex). However, it differs from the assemblages observed in MSA II or in early MIS 5 industries, where triangular production is a notable occurrence (e.g., Unit F at Florisbad^15,82,83^, Phase M3 at Blombos Cave^84^, Member 4 WA at Border Cave^85^, MSA II at Klasies River^86^, and MSA-Mike at Diepkloof Rock Shelter^87^). Despite its MIS 5 age, more pertinent technological parallels may be found in assemblages frequently grouped under the heuristic label of EMSA. These assemblages are often characterised by a stronger emphasis on laminar and flake production, as documented in Units N to G at Florisbad^15^, in Excavation 2 (MU3 to MU8) at Wonderwerk Cave^88^, in Phases L and K at Elands Bay Cave^56^, and in LC-MSA at Pinnacle Point 13B^89^. Given the open-air context and limited artefact density, the assemblage is not interpreted here as reflecting a specific site function (e.g., workshop, residential locality). Instead, technological characteristics are used comparatively, to situate Baden-Baden 2 within broader patterns of MSA variability, while recognising that functionally distinct activities may be conflated within the recovered assemblage.

*Chronological context of the Baden-Baden 2 industry*. As stated, the Baden-Baden 2 technology may be closer to that of the nearby Middle Pleistocene lithic assemblages found at Florisbad, although the latter are dated to > 130 ka and in part fall under the label of EMSA^15,18,33,34^. In regional syntheses, the EMSA is generally placed broadly between ~ 300 − 130 ka (approximately MIS 8–6), functioning in practice as a shorthand for MSA assemblages that pre-date better-defined later entities (e.g., Still Bay)^3,90^. Importantly, these same syntheses explicitly acknowledge that the EMSA label remains provisional and requires further clarification, particularly with respect to its internal variability, chronological resolution, and technological coherence.

**Fig. 11:**
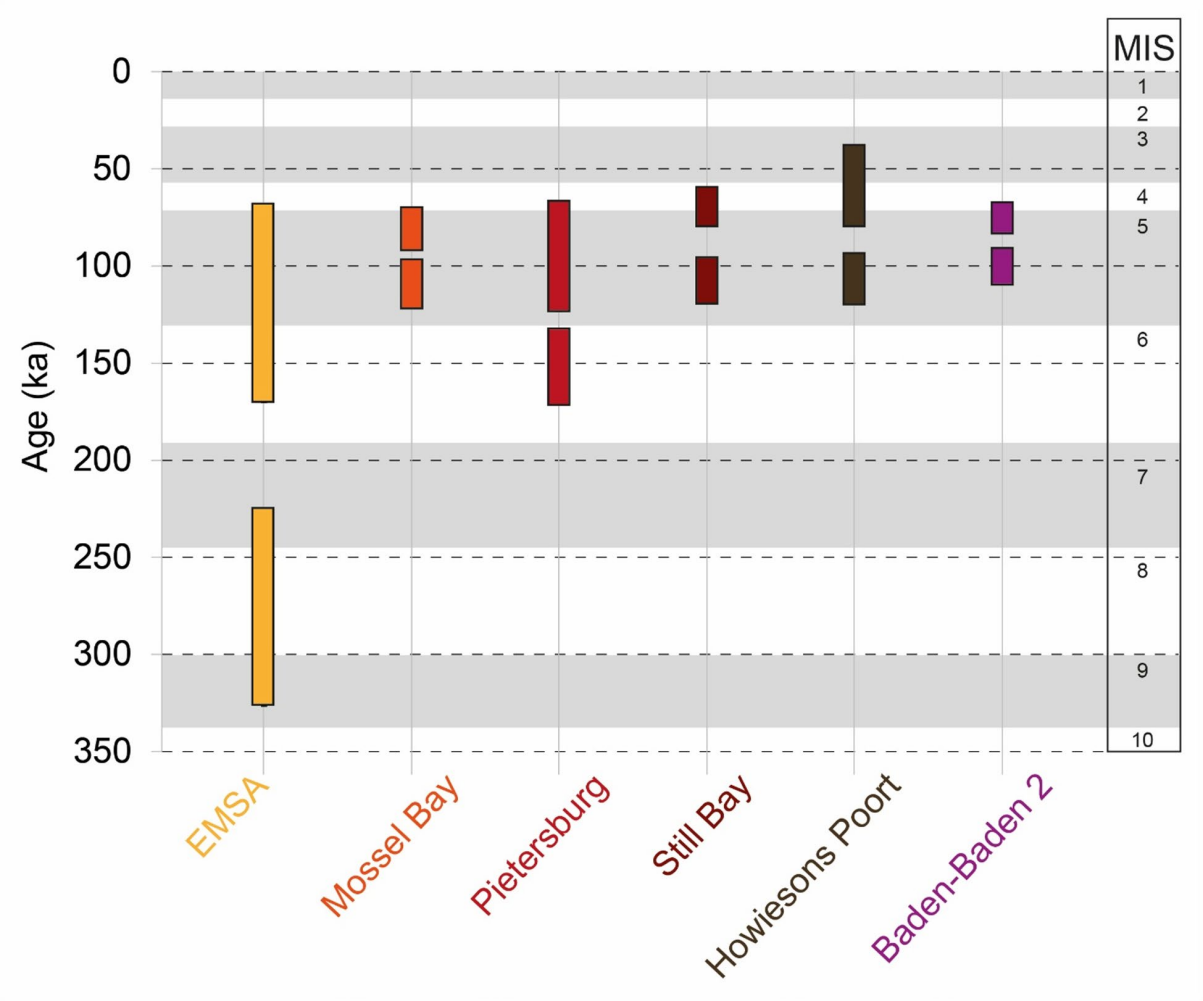
Comparison of the chronological range of Baden-Baden 2 with those of other lithic assemblages in South Africa spanning MIS 9 to 4. The Mossel Bay, Pietersburg, Still Bay, and Howiesons Poort assemblages and ages are according to Lombard et al.^57^ and references therein. The EMSA ages are cited in the text.

By comparing the ages of South African lithic assemblages dated to MIS 8 − 4, a complex chronological context emerges. The EMSA assemblage at Florisbad was dated using OSL to 279 ± 47 ka (Unit P) and 281 ± 73 (Unit O)^18^. It should be noted that Kuman et al.^15^ define the assemblage from Unit M, dated to 157 ± 21 ka, as MSA, and thus distinct from the underlying EMSA of Units P-O-N based on technology. A similar temporal range for the EMSA was obtained for Elands Bay Cave (Liam Unit), with ages of 231 ± 20 ka (OSL on quartz), 249 ± 24 ka (IR_50_ on feldspars), and 227 ± 22 ka (pIRIR_290_ on feldspars)^22^. At Klipfonteinrand 1, layer PBS was dated to 166 ± 13 and 146 ± 16 ka using OSL^23^. However, several studies have shown that lithic assemblages called EMSA may be younger than 130 ka. This includes one thermoluminescence age obtained on a burnt quartzite at Elands Bay Cave of 83 ± 14 ka (Keva Unit)^22^; at Varsche Rivier 003, three pIRIR_225_ ages obtained on feldspars ranging from 90 ± 5 to 81 ± 5 ka (pre-Still Bay layers I-08 and I-09)^91^, three U-series ages obtained on ostrich eggshell fragments ranging from 90.7 ± 0.7 ka to 74.5 ± 1.3 ka (I-09)^91^, and one OSL age of 61 ± 4 ka (layer II-07)^92^; two OSL ages at Klipfonteinrand 1 of 91 ± 5 ka and 80 ± 16 ka (layer GGLBS)^23,93^; at Kathu Pan 6, one OSL age of 95 ± 6 (Bed 11)^21^; and an age range obtained from feldspar in two samples measured using the pIRIR_225_ protocol, bracketing the archaeological assemblages at Jojosi 1 from 139 to 106 ka^58,94^. These ages are comparable to those obtained for Baden-Baden 2, considering the 1σ error range (Fig. 11).

The ages presented here suggest that assemblages commonly labelled as EMSA appear toward the end of the Middle Pleistocene and continue well into MIS 5. In this context, Baden-Baden 2 falls within the broad temporal range of several MIS 5 and 4 assemblages in South Africa that have been variably classified as EMSA, Mossel Bay, Pietersburg, Still Bay, and Howiesons Poort (Fig. 11). The substantial chronological overlap among these assemblages (e.g., Wurz^95^ for the Pietersburg) indicates that the EMSA cannot be used as a reliable chronological marker for defining MSA assemblages older than ~ 130 ka. Rather, this overlap likely reflects regionally asynchronous trajectories of technological change, compounded by the difficulty of delimiting lithic industries during MIS 8–5—a poorly resolved interval that remains in need of more systematic and comparative investigation across southern Africa.

The Baden-Baden 2 lithic assemblage falls within the larger issue of identifying MSA cultures before the onset of MIS 5, a period marked by a discontinuity in the production of broad types of blanks and characterised in particular by the emergence of triangular blank production. Baden-Baden 2 may represent a late manifestation of assemblages traditionally labelled as EMSA in the Free State, preceding the appearance of more internally homogeneous lithic industries during late MIS 5 and MIS 4, as documented for instance at Rose Cottage Cave^96,97^ and Lovedale^26,29,98^ in the Free State. Used here descriptively rather than as a formal technocomplex, Baden-Baden 2 currently provides a key technological, spatial, and temporal reference point for the western Free State. Ongoing detailed analysis of the lithic assemblage aims to better define the nature of the site and to situate it within broader patterns of technological variability during MIS 5 in South Africa, rather than within a rigid cultural or chronological framework.

## Methods

### Archaeological excavations

Archaeological excavations took place in July 2023 and June-July 2024. We laid out 1 × 1 m squares, called Units, excavated by 20-cm arbitrary spits, called Levels. During the 2023 season, a 2 × 1 m trench (Unit 1 and Unit 2) was placed near the top of the dune, above the elevation at which artefacts appeared on the ground, with the aim of finding them embedded in the original sedimentary matrix. Another 2 × 1 trench (Unit 3 and Unit 4) was laid out in the eroded area on the southeastern flank of the dune to verify whether the surface artefacts were in primary deposition. In 2024, a 2 × 4 m trench (Units 5 to 12) was laid out one metre to the northwest of Unit 1 to expand the horizontal view of the site and the lithic assemblage. The trenches were excavated using small tools following the 20-cm arbitrary levels, except for Levels 5–7 in 2024, which were set at 10 cm to account for the richness of artefacts. Sediments were sieved through 4 mm and 2 mm meshes to recover small artefacts and stone chips. The 2 mm mesh did not produce artefacts. Trenches, artefacts, and sediment samples were piece-plotted using a total station tied to a grid system developed for the site. A large proportion of surface artefacts were also plotted and collected. No faunal material was found at the site.

### FTIR spectroscopy

The sediment samples collected in 2023 for MS (*n* = 16) were analysed using FTIR spectroscopy in order to determine their composition. Samples were sieved through a 53 μm mesh to remove the coarse quartz fraction, which hinders a proper evaluation of other silicate phases, such as the phyllosilicates^17^. About 10 mg of the fraction smaller than 53 μm were ground using an agate mortar and pestle and mixed with 20 mg of FTIR-grade KBr (Sigma-Aldrich). Each mixture was pressed into a 7-mm pellet by applying 2 tons of pressure using a benchtop mini-press (Specac). Pellets were analysed using a Themo Scientific Nicolet iS5 infrared spectrometer in the 4000 –400 cm^− 1^ spectral range at 4 cm^− 1^ resolution and in 32 scans. Phase identification was performed with OMNIC v. 9.13 based on the infrared spectral library of Archéosciences Bordeaux^99^.

### Micromorphology of sediments

Micromorphology blocks (*n* = 11) were collected next to the luminescence samples (Fig. 3) in order to determine the formation and post-depositional processes of the site. Blocks of intact sediment were carved out of sections, jacketed with plaster of Paris bandages, and shipped to France (2023) and Spain (2024) for processing at the laboratory. Blocks were oven-dried over several weeks and embedded in a mixture of polyester resin and acetone under vacuum. Once solid, the blocks were cut into 6 × 10 cm or 7 × 14 cm chips using a rock saw. The chips were mounted on glass slides and polished to a thickness of 30 μm. Thin sections were observed at magnifications ranging from 25X to 400X and described according to standard literature^100–102^. Since similar depositional units occurred repeatedly throughout the sedimentary context, we decided to group the micromorphological samples under different microfacies types (MFT), according to the description given by Flügel^103^: “the total of all sedimentological and paleontological data which can be described and classified from thin sections, peels, polished slabs or rock samples”.

### Magnetic properties

Sediment samples were collected every 5–10 cm from the north section of Unit 1 (*n* = 16) in order to measure their volume magnetic susceptibility (MS) and anhysteretic remanent magnetisation (ARM), with the aim of identifying changes in sediment supply (which may imply breaks in the sedimentation or erosional events) and the concentration of magnetic minerals (such as magnetite) in sediments, respectively^28^. Samples for MS were prepared by transferring ~ 25 g of sediment into plastic holders, which were then placed in the MS reader. Two analyses were conducted for each sample and the results were averaged. Low field, bulk magnetic susceptibility was measured with a MFK1-FA (AGICO) at a frequency of 976 Hz, after weighting the samples. Samples for ARM were prepared by mixing ~ 4 g of sediment with 50% sodium silicate inside plastic holders, a step necessary to stabilise unconsolidated samples during analysis. The mass-normalised ARM was obtained by dividing the magnetic moment of the sample by its mass (expressed in kg). The ARM was designed to isolate the fine-grained magnetite concentration of the mass normalised samples by using a 100 mT AF field and a biasing 0.1 mT DC field as a function of stratigraphic depth. Measurements were carried out with a 3-axis, 4.2 cm-access SQUID magnetometer (755–4 K Superconducting Rock Magnetometer, “Helium-free”, 2G Enterprises).

### Image analysis of sand grains

Sand grains in the OSL samples were analysed using a Malvern Morphologi G3 particle characterisation system to characterise the shape, form, and size of particles, and thus provide information on their degree of bleaching for luminescence dating. Rounded particles are more likely to be the result of long transport by wind, suggesting that the previous accumulated luminescence signal was completely reset. Sand grains were dispersed over a glass plate by air injection and high-resolution greyscale images of the studied area were collected. The scanned area and the optics were selected for each size fraction to ensure a representative sample was obtained. The sample volumes were 14 mm^3^ for all samples, and the fraction range was 6.5–420 μm. After each analysis, joined particles, non-minerals (i.e., dust) and particles < 70 μm were manually excluded. The size parameters used in the analysis were circle equivalent diameter, length, and width, and the targeted shape parameter was the high sensitivity circularity (HSC), which indicates the similarity of a particle to a circle. Values range from 0 (extremely narrow rod) to near 1 (perfect circle).

### Luminescence dating

Nine sediment samples were collected in lightproof bags from the two sections (Fig. 3); five samples from Units 1 and 2 (Levels 3, 4, 5, and 6) and four from Units 5 and 6 (Levels 1, 5, 6, and 8), including one modern reference to assess the degree of bioturbation and bleaching in the recent sediment. Each sampling location, a few square centimetres across, was then enlarged into a hole to conduct in-situ gamma spectrometry measurements using a portable gamma-ray spectrometer connected to a LaBr probe (Inspector 1000, Canberra). The sediment from the hole was kept to measure the water content (water/dry weight of sediment %) and the U, Th, and K content at the laboratory using a gamma-ray spectrometer equipped with a high-resolution, broad energy Ge (BEGe) detector. The gamma spectrometry hole was further enlarged to carve micromorphology blocks.

The sediment samples were prepared and analysed at the luminescence laboratory of Archéosciences Bordeaux (Pessac, France) under subdued red light and controlled conditions in order to prevent light exposure. Samples were wet-sieved at 100 and 120 μm and chemically treated using HCl (10%) to dissolve the carbonates and H_2_O_2_ (30%) to degrade the organic matter. Quartz was separated from heavy minerals (at ρ = 2.72 g/cm^3^) and feldspars (at ρ = 2.62 g/cm^3^) using density separation with a heavy liquid (lithium heteropolytungstate or sodium polytungstate). The quartz fraction was purified using H_2_SiF_6_ (31%) over one week to remove the possible remaining feldspars and rinsed with HCl (10%).

The equivalent doses were measured on 100–120 μm grain-size quartz loaded on 150 μm holes-disks. The single grain (SG) measurements were conducted on a Risø OSL/TL reader using the protocol displayed in Table 5. A green laser was used to measure 1000 grains and the signal was detected using Hoya U340 filters, and integrated using the first 0.07 s and the background subtracted using the last 0.25 s. The following criteria were applied for data selection: a recycling ratio limit of 10%; a recuperation < 5% of the natural signal; a maximum test dose error of 10%; and a test dose signal > 3 sigma above background. The dose response curves (DRC) were obtained using an exponential function. Dose recovery tests (DRT) were performed on 300–500 grains for each sample: the disks were bleached two minutes in a solar simulator (Hönle 500) and a given dose of 2 Gy (BAD-MOD), 75 Gy (BAD1, 2, 3, 5, 6, 7, 8), and 200 Gy (BAD4) was applied before applying the single aliquot regeneration (SAR) protocol^104^ in Table 5. The luminescence data were treated using Analyst v. 4.57^105^.

Considering the extensive bioturbation at the site, the Finite Mixture Model (FMM^41,42^) was applied using the luminescence package available on R^106^. Following the recent study of Peng et al.^107^, we use the approach that vary the σ_b_ to fit the FMM (here, from 0.1 to 0.6, with increments of 0.05, and up to *k* = 9), and select the combination of σ_b_ and *k* that yielded the lowest BIC score, and for comparison purpose, we ran the FMM with a σ_b_ = 0.20 (based on the mean value obtained for well-bleached and undisturbed sand^44^). In both cases, the fit with the lowest BIC score was used for age calculation.

**Table 5:** OSL single grain (SG) SAR protocol applied to quartz.

Step	Treatment
1	Given dose
2	Preheat (240 °C for 10 s)
3 (Lx)	SG green laser stimulation for 1 s at 125 °C
4	Given test dose
5	Cutheat (200 °C for 0 s)
6 (Tx)	SG green laser stimulation for 1 s at 125 °C
7	Optical draining (blue stimulation for 120 s at 280 °C)
8	Return to 1

The dose rate was calculated using both in situ and laboratory measurements. The in-situ gamma dose rate data were processed using the “threshold” technique^108^. The sediment samples were dried in an oven for at least 7 days and the difference between the wet and the dry weight was used to calculate the water content. These samples were then sealed in a container and analysed using laboratory gamma-ray spectrometry. The alpha and beta dose rates were derived using the conversion factors of Guérin et al.^51^. The cosmic dose rate was evaluated from current sample depth using the equations of Prescott and Hutton^52^.

### Plant biomarkers

Thirteen sediment samples were collected at regular intervals from the north section of Unit 6 (Fig. 3), of which 11 were analysed. In the laboratory, samples were sieved and then homogenised by grinding using a mortar and pestle. Extraction of *n*-alkanes followed a modified protocol from Wang et al.^72^. 16 g of sediment from each sample were used for extraction with an accelerated solvent extractor (ASE-200, Dionex) at 100 bar and 100 ˚C using a 9:1 (v = v) mixture of dichloromethane (DCM) and methanol. The *n*-alkanes were subsequently separated by silica gel column chromatography using activated silica gel and hexane and further separated using silver nitrate (AgNO_3_) coated silica gel. Individual *n*-alkane homologues were identified with an Agilent 6890 N gas chromatograph equipped with a Restek XTI-5 capillary column (30 m x 320 μm x 0.25 μm) based on the comparison of their retention times with a standard containing the *n*-alkane homologues *n*-C_20_, *n*-C_24_, *n*-C_28_, *n*-C_32_, *n*-C_36_, *n*-C_38_, and *n*-C_40_ of known concentration. All the samples were measured in triplicates. The following GC-FID temperature programme was used: the oven was maintained for 1 min at an initial temperature of 50 °C, then the temperature was increased to 150 °C (5 min hold) before a further increase to 330 °C (5 min hold).

The carbon preference index (CPI) was calculated using the following formula:

CPI_25−35_ = [(C_29_+C_31_) + (C_27_+C_29_+C_31_+C_33_+C_35_)] / [2x(C_26_+C_28_+C_30_+C_32_+C_34_)]

The average chain length (ACL) was calculated using the following formula:

ACL_25−35_ = (25 x C_25_ + 26 x C_26_ + 27 × C_27_ + 28 x C_28_ + 29 × C_29_ + 30x C_30_ + 31 × C_31_ + 32 x C_32_ + 33 × C_33_ + 34 x C_34_ + 35 x C_35_) / (C_25_ + C_26_ + C_27_ + C_28_ + C_29_ + C_30_ + C_31_ + C_32_ + C_33_ + C_34_ + C_35_).

The Norm_31_ is calculated as: Norm_31_ = C_31_ / (C_29_ + C_31_) and Norm_33_ as: Norm_33_ = C_33_ / (C_29_ + C_33_).

Terrestrial-sourced *n*-alkane homologues of sufficient concentration (i.e. *n*-C_27_, *n*-C_29_, *n*-C_31_, and *n*-C_33_) were analysed using gas chromatography-isotope ratio mass spectrometry (GC-IRMS) for δD and δ^13^C at the Leibniz Laboratory for Radiometric Dating and Stable Isotope Research at Kiel University. Samples were measured on an Agilent 6890 gas chromatograph equipped with a Gerstel KAS 4 PTV injector and an Agilent DB-5 capillary column (30 m x 250 μm x 0.25 μm) coupled to a Thermo Scientific MAT 253 isotope ratio mass spectrometer (IRMS). Following GC temperature programme was used for the analysis of carbon and hydrogen isotopes: 50 °C for 5 min, with 40 °C/min to 240 °C, with 20 °C/min to 280 °C, with 10 °C/min to 325 °C, 325 °C for 20 min. Depending on the *n*-alkane concentration, 10 µl and 20 µl of each sample for δ^13^C and δD, respectively, was injected 2–4 times in order to achieve a statistically robust analytical error for each *n*-alkane homologue. To allow large volume injections (LVI), the injector was used in solvent vent mode. The H3 + factor during the measurement period was 10.34 ± 0.19 ppm/nA (*n* = 9). The δD and δ^13^C values are reported relative to the Vienna Standard Mean Ocean Water (‰ VSMOW) and Vienna Pee Dee Belemnite (‰ VPDB) scales using Arndt Schimmelmann’s A7 reference mixture from 2017.

### Lithic analysis

A sample of 467 items was selected from the total assemblage of 1153 lithic artefacts based on the identification of technological features enabling the reconstruction of the operational sequences (e.g., modalities, chronology of removal negatives). The sample includes 233 artefacts collected from the surface and 234 artefacts recovered from 12 excavation Units. Of the excavated artefacts, 65 were found in a secondary context (Unit 3). Given the low count of artefacts, the lithic study focused primarily on a qualitative analysis. This was carried out through a typo-technological approach. The descriptive terminology adheres to the glossary proposed by Inizan et al.^109^. Retouched and shaped artefacts are classified according to the criteria established by Bordes^110^, while the identification of technological features follows the framework proposed by Conard et al.^60^ and Boëda^61,111^. In addition, the criteria used to examine and establish the stages of the reduction sequences are based on the methodological principles outlined by Geneste^112^.

## Supplementary Information

Below is the link to the electronic supplementary material.

Supplementary Material 1

Supplementary Material 2

Supplementary Material 3

## Data Availability

All data needed to validate the conclusions in the paper are present in the paper and/or the Supplementary Information materials.
