# Supplementary material for: A Middle Stone Age occupation identified at Baden-Baden in the grasslands of the Free State, South Africa
Source: Sci Rep. 2026 Apr 6;16:12027. doi: 10.1038/s41598-026-43246-9 (PMC13068903; doi:10.1038/s41598-026-43246-9)
Supplement: Supplementary file 3 — Supplementary Material 3 [file 41598_2026_43246_MOESM3_ESM.pdf]

A Middle Stone Age occupation identified at Baden-Baden in the grasslands of the Free State, South Africa

Maïlys Richard[1,2,*], Beatrice Bin[1,3], Benoit Longet[1,3], Michaela Ecker[4,5], Nils Andersen[6], Will Archer[7,8,9,10], Myra Gohodzi[8], Sharon Holt[8], C. Britt Bousman[11], Michael B. Toffolo[1,3,12]

[1]Archéosciences Bordeaux, UMR 6034 CNRS-Bordeaux Montaigne University, Esplanade des Antilles, 33607 Pessac, France

[2]Department of Early Prehistory and Quaternary Ecology, University of Tübingen, Burgsteige 11, 72070 Tübingen, Germany

[3]Geochronology and Geology Programme, National Research Centre on Human Evolution (CENIEH), Paseo Sierra de Atapuerca 3, 09002 Burgos, Spain

[4]Institute of Prehistoric and Protohistoric Archaeology, Kiel University, Johanna-Mestorf-Straße 2-6, 24118 Kiel, Germany

[5]Archaeology Department, McGregor Museum, Atlas Street, 8301 Kimberley, South Africa

[6]Leibniz Laboratory for Radiometric Dating and Isotope Research, Kiel University, Max-Eyth-Straße 11-13, 24118 Kiel, Germany

[7]Max Planck Partner Group, Department of Archaeology and Anthropology, National Museum Bloemfontein, 9301 Bloemfontein, South Africa

[8]Florisbad Quaternary Research Station, National Museum Bloemfontein, 9301 Bloemfontein, South Africa

[9]Department of Geology, University of the Free State, Zastron Street, 9301 Bloemfontein, South Africa

[10]Department of Anthropology, The George Washington University, 2110 G St. NW Washington, DC 20052, USA

[11]Department of Anthropology, Texas State University, 601 University Drive, San Marcos, TX 78666-4684, USA

[12]Department of Plant Sciences, University of the Free State, Zastron Street, 9301 Bloemfontein, South Africa

*Corresponding author. Email: mailys.richard@cnrs.fr

Supplementary Information

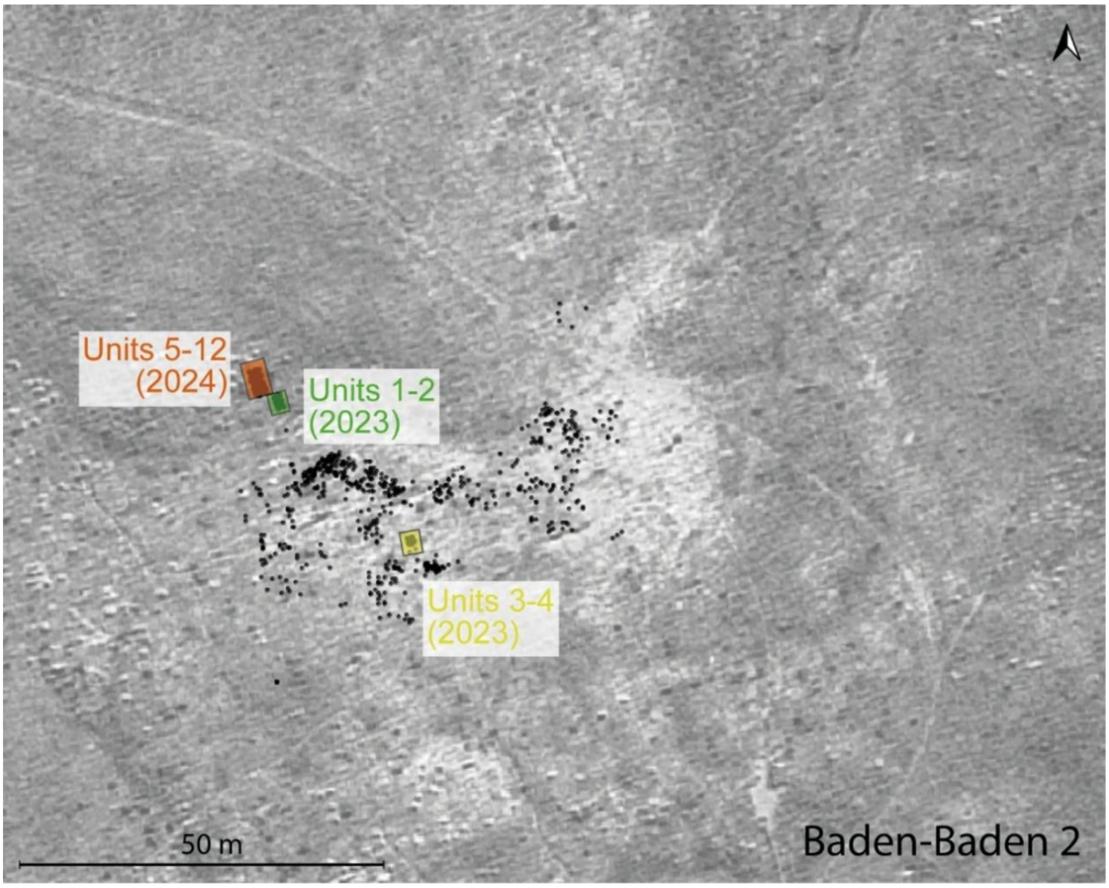

Figure S1. Aerial view of Baden-Baden 2 showing the location of excavation Units and plotted artefacts (black dots).

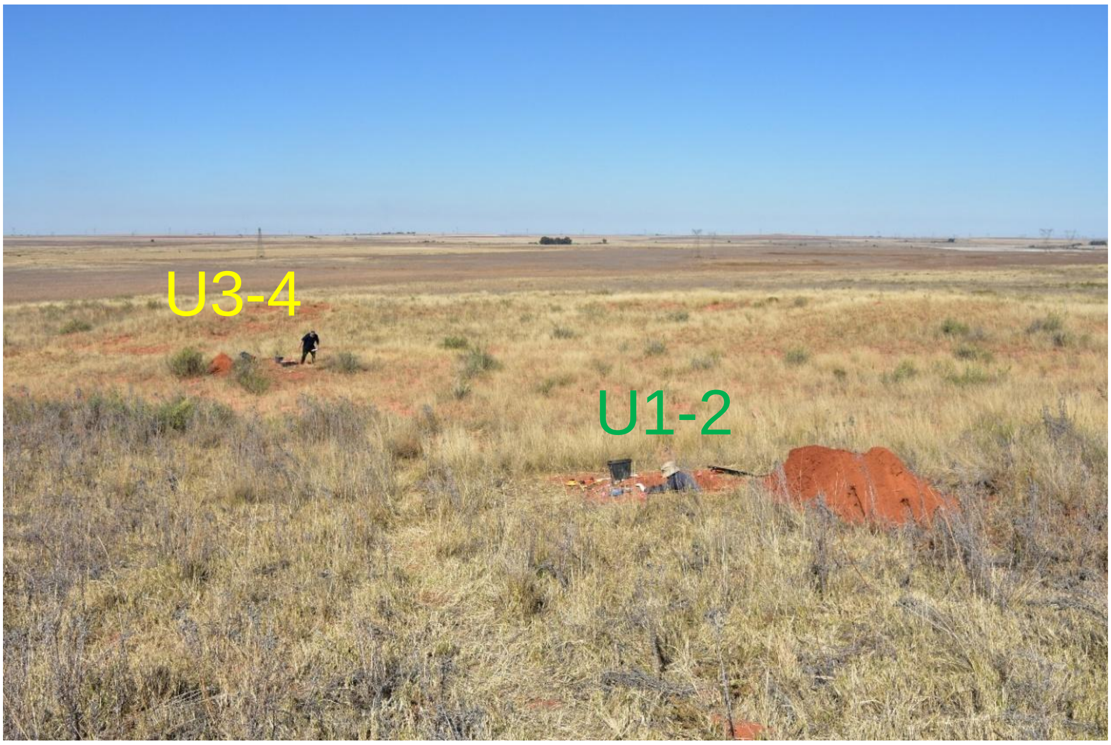

Figure S2. Units 1-2 and 3-4 during the 2023 excavation.

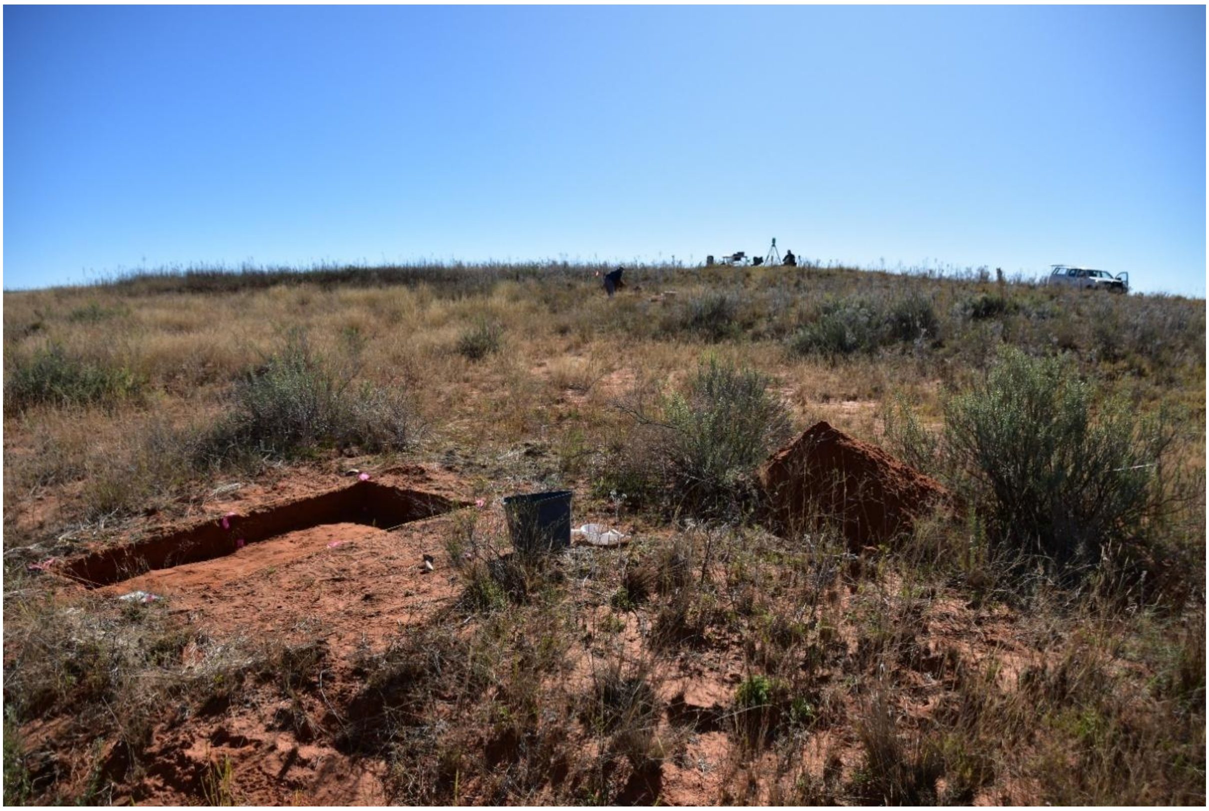

Figure S3. Units 1-2 during the 2023 excavation.

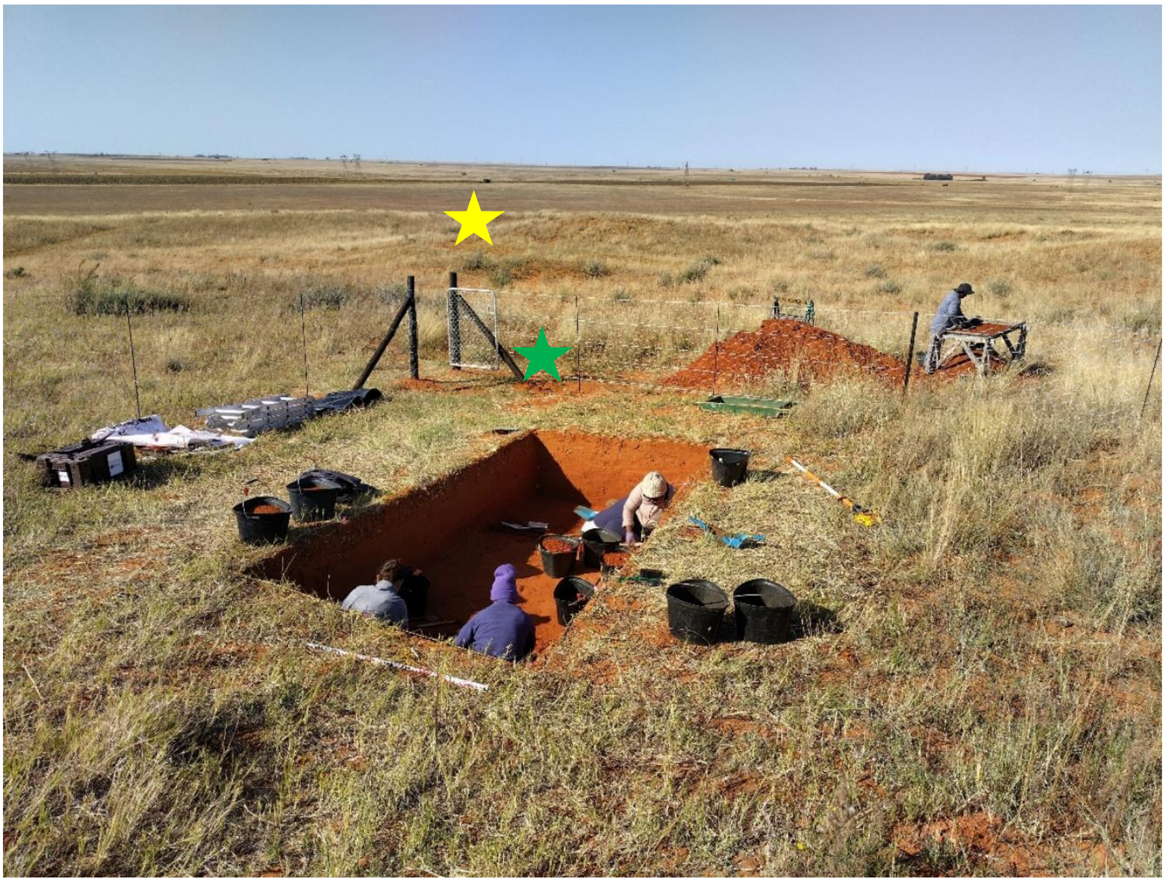

Figure S4. Units 5-12 during the 2024 excavation. Units 1-2 and 3-4 (2023) are marked by a star.

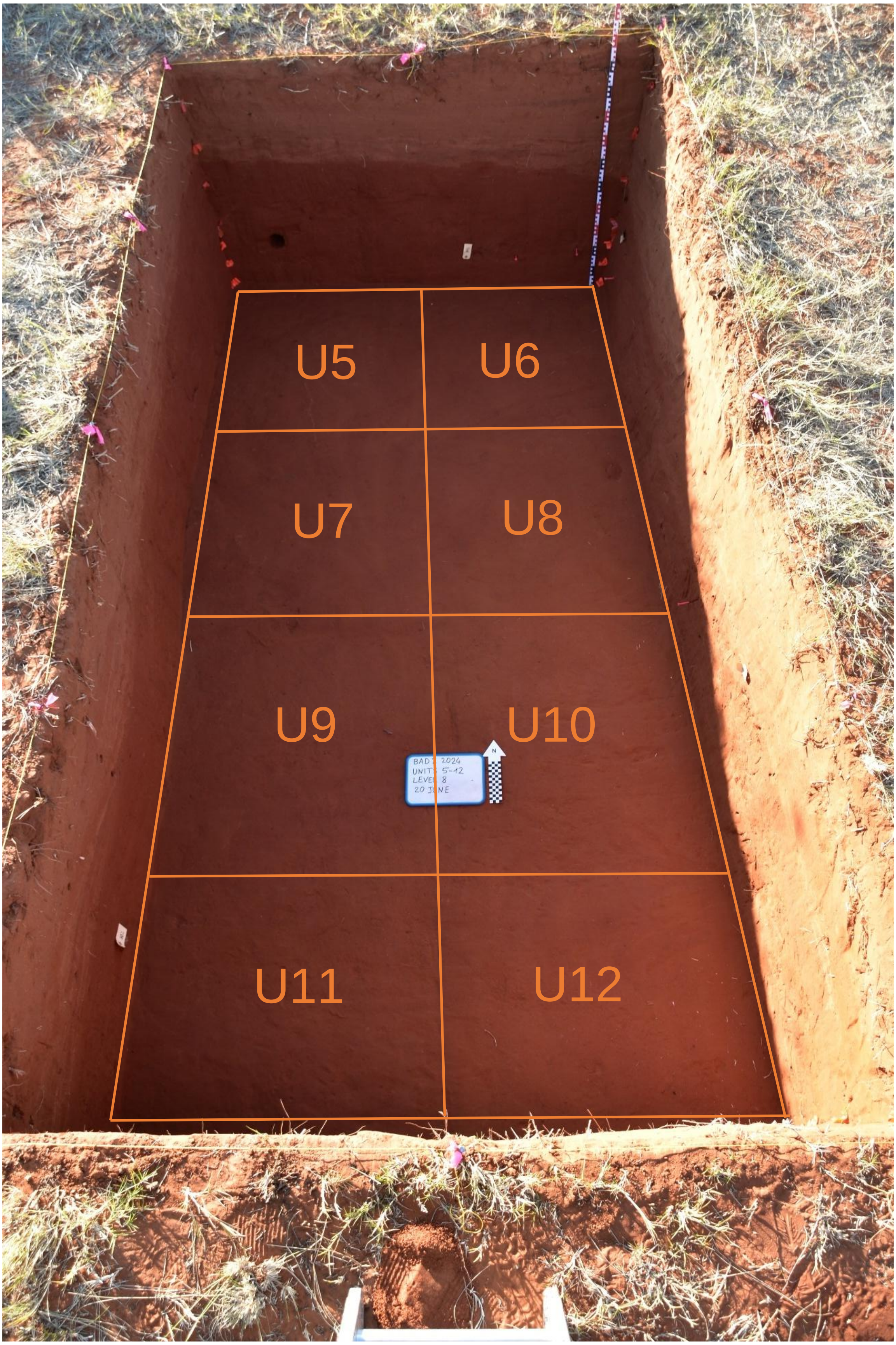

Figure S5. View of Units 5-12 during the 2024 excavation, showing the top of Level 8.

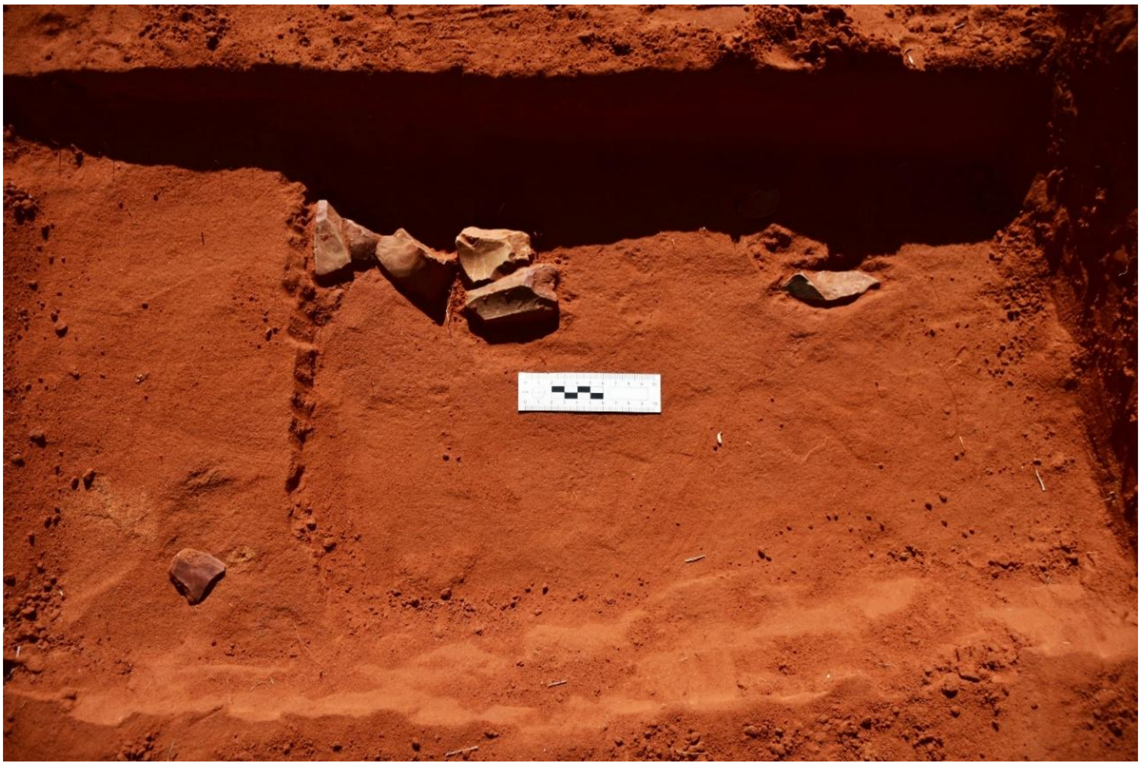

Figure S6. Example of a cluster of lithic artefacts lying horizontally on an occupation surface at ~100 cm depth in Unit 10.

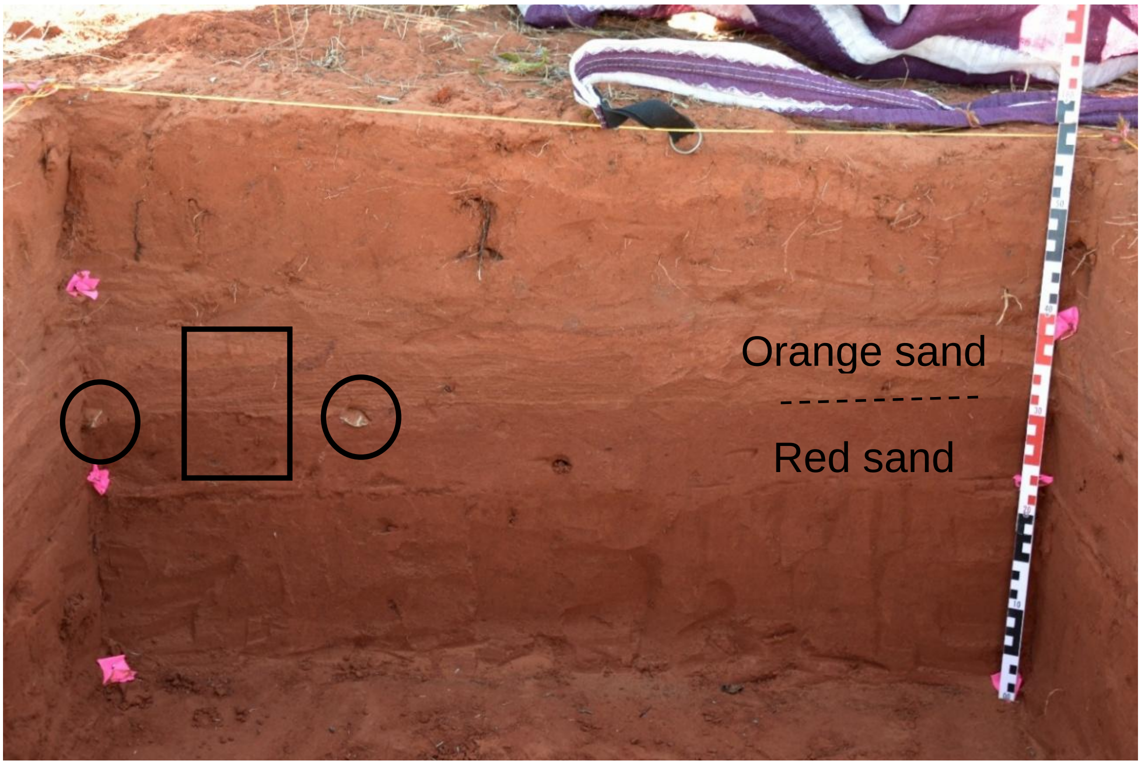

Figure S7. North section of Unit 3 at the end of the 2023 season showing the location of micromorphology block 3-1 (rectangle) and artefacts (circles) at the contact between orange and red sand, sealed by thin beds of modern colluvium. The dashed line marks the boundary between orange sand and red sand.

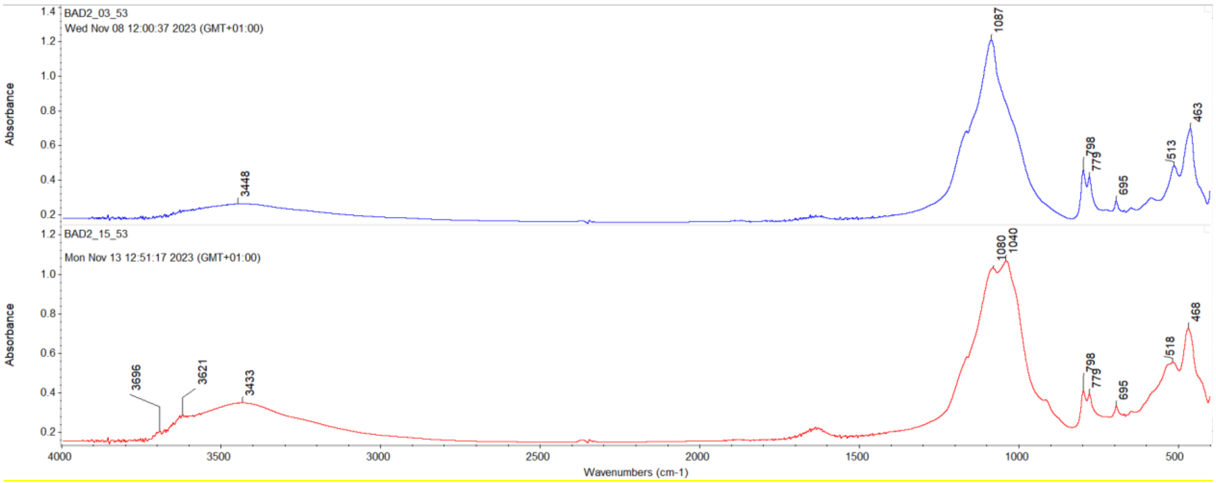

Figure S8. Representative FTIR spectra of orange sand (top) and red sand (bottom).

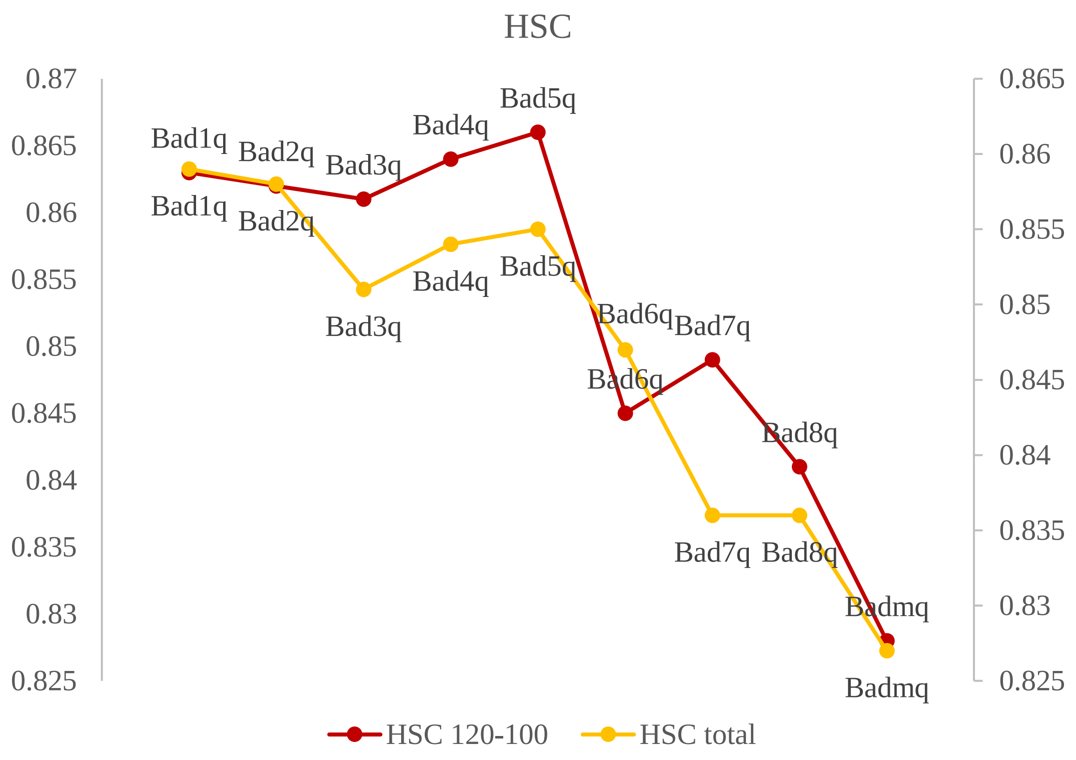

Figure S9. Comparison between HSC mean values of 120-100 µm fraction and HSC mean values of all fractions.

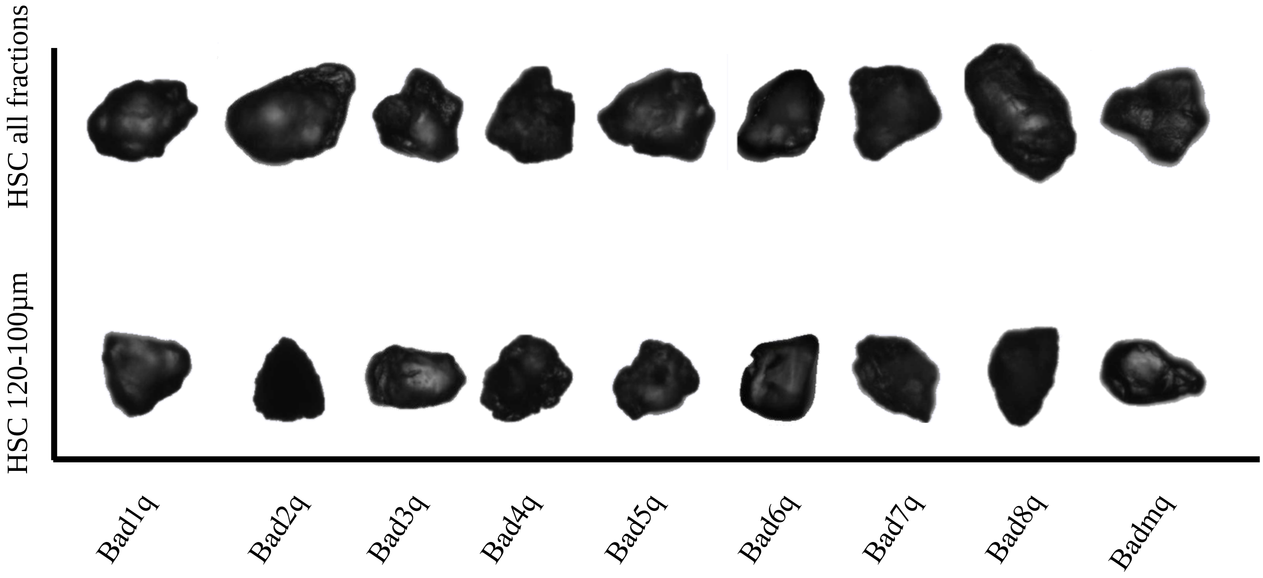

Figure S10. Comparison between HSC mean values of all fractions and HSC mean values of 120-100 µm fraction.

Table S1. Mean values of HSC and number of particles of fraction 120-100 µm.

Sample	Mean value	Min. value	Max. value	N. of particles
**BAD1q**	0.863	0.736	0.945	37
**BAD2q**	0.862	0.680	0.955	249
**BAD3q**	0.861	0.702	0.981	190
**BAD4q**	0.864	0.615	0.962	360
**BAD5q**	0.866	0.673	0.961	213
**BAD6q**	0.845	0.641	0.955	139
**BAD7q**	0.849	0.737	0.919	25
**BAD8q**	0.841	0.700	0.929	58
**BADmq**	0.828	0.750	0.898	40

Table S2. MFTs identified at Baden-Baden 2. The terminology follows Stoops[1].

MFT name	Description	Process	Sample
Bioturbated sand	**c/f related distribution:** chito-gefuric to enaulic. **Microstructure and voids:** intergrain microaggregate with abundant channels, chambers, and planes. **Coarse fraction:** sand-sized sub-angular quartz grains (dominant); sand-sized and silt-sized sub-rounded quartz grains (few); silt-sized sub-angular quartz grains (few); gravel-sized sub-angular and sub-rounded quartz grains (very few); sand-sized sub-angular felspar grains (few); sand-sized and silt-sized sub-rounded magnetite grains (few); sand-sized and silt-sized sub-angular magnetite grains (few); gravel-sized hornfels fragments (very few). **Fine fraction:** orange to reddish clay with stipple-speckled, striated, and grano- and poro-striated b-fabric. **Pedofeatures:** coatings, hypo-coatings, and nodules of Fe-Mn hydroxide; plant remains in channels.	Aeolian deposition<br>Bioturbation	0, 1-4, 8, 3-1
Cross-bedded sand	**c/f related distribution:** chito-gefuric to enaulic. **Microstructure and voids:** intergrain microaggregate with few channels, planes, and chambers. **Coarse fraction:** sand-sized sub-angular quartz grains (dominant); sand-sized and silt-sized sub-rounded quartz grains (few); silt-sized sub-angular quartz grains (few); gravel-sized sub-angular and sub-rounded quartz grains (very few); sand-sized sub-angular felspar grains (few); sand-sized and silt-sized sub-rounded magnetite grains (few); sand-sized and silt-sized sub-angular magnetite grains (few); gravel-sized hornfels fragments (very few). **Fine fraction:** reddish clay with striated to grano- and poro-striated b-fabric. **Pedofeatures:** coatings and hypo-coatings of clay; nodules of Fe-Mn hydroxide; nodules of Fe-Mn hydroxide; fragments of transported clay; plant remains in channels.	Aeolian deposition<br>Sheetwash<br>Bioturbation	5-7, 9
Modern sheetwash	**c/f related distribution:** chito-gefuric. **Microstructure and voids:** intergrain microaggregate with few channels, planes, and chambers. **Coarse fraction:** sand-sized sub-angular quartz grains (dominant); sand-sized and silt-sized sub-rounded quartz grains (few); silt-sized sub-angular quartz grains (few); gravel-sized sub-angular and sub-rounded quartz grains (very few); sand-sized sub-angular felspar grains (few); sand-sized and silt-sized sub-rounded magnetite grains (few); sand-sized and silt-sized sub-angular magnetite grains (few); gravel-sized hornfels fragments (very few). **Fine fraction:** orange clay with stipple-speckled b-fabric. **Pedofeatures:** coatings and hypo-coatings of clay; nodules of Fe-Mn hydroxide.	Aeolian deposition<br>Sheetwash<br>Bioturbation	3-1 (Unit 3)

Table S3. Magnetic susceptibility (MS) and anhysteretic remanent magnetisation (ARM) values from Unit 1 at Baden-Baden 2.

Sample	MS	ARM
MS01	4.30E-07	1.40E-01
MS02	4.16E-07	1.32E-01
MS03	3.68E-07	1.07E-01
MS04	4.19E-07	1.51E-01
MS05	4.20E-07	1.48E-01
MS06	4.04E-07	1.49E-01
MS07	4.34E-07	1.42E-01
MS08	4.41E-07	1.43E-01
MS09	4.26E-07	1.47E-01
MS10	4.23E-07	1.42E-01
MS11	4.40E-07	1.07E-01
MS12	4.31E-07	1.31E-01
MS13	4.35E-07	1.33E-02
MS14	4.33E-07	1.29E-01
MS15	4.08E-07	1.25E-01
MS16	4.28E-07	1.39E-01

Table S4. Statistics on the selected single grains and dose recovery test (DRT) results. [1]Total number of individual quartz grains measured using the SAR protocol; [2]Number of grains that provided a luminescence signal passing the following criteria: recycling ratio ≠ 1 ± 10%; recuperation < 5%; error on the test dose <10%; T_n signal > 3σ above background; [3]Among the grains that passed criteria, those for which no intersection was reached, those for which the L_n/T_n signal was higher than the highest regenerated point (extrapolated grains), those for which the D_e was higher than two times D_0[2], and those for which a $D_e < 0$ was obtained. [5]The dose recovery test (DRT) was conducted for each sample on 300-500 grains; The given dose is 75 Gy except for BAD-04 (200 Gy) and the modern sample BAD-MOD (2 Gy). The calculated DRT value corresponds to the mean recovered value/given value ± the standard deviation in % of all accepted grains, with the corresponding overdispersion (OD) value.

	BAD1	BAD2	BAD3	BAD4	BAD5	BAD6	BAD7	BAD8	BAD-MOD
Number of grains measured[1]	1000	1000	1000	1000	1000	1000	1000	1000	1000
Number of grains that passed criteria[2]	139	147	169	155	133	149	127	139	54
Number of rejected grains[3]	37	52	62	36	55	43	30	49	5
- No intersection	13	25	26	21	25	15	15	20	3
- Extrapolated grains	6	3	3	2	6	0	1	0	0
- Saturated grains (De > 2*D_0)	18	24	32	28	24	28	14	29	0
- Negative D_e	0	0	1	0	0	0	0	0	2
Accepted grains[4]	102	95	107	104	78	106	97	90	35
DRT[5]	1.05 ± 21%	1.00 ± 18%	1.08 ± 18%	0.85 ± 17%	1.07± 29%	1.03 ± 19%	1.08 ± 18%	1.02 ± 19%	1.13 ± 13%
OD (%)	19%	14%	9%	11%	24%	10%	10%	13%	4%

a) 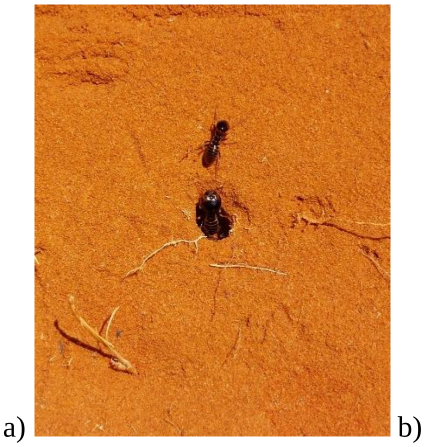 b) 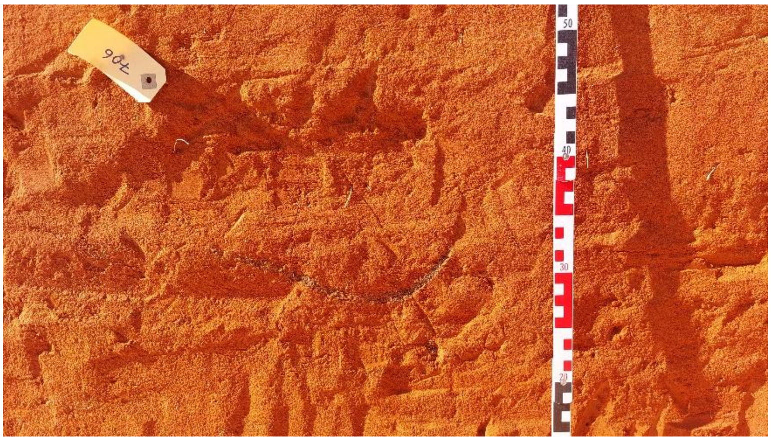

Figure S11. Termites in activity, top of the sequence (a), and channel filled with sediment (b), 2024 trench.

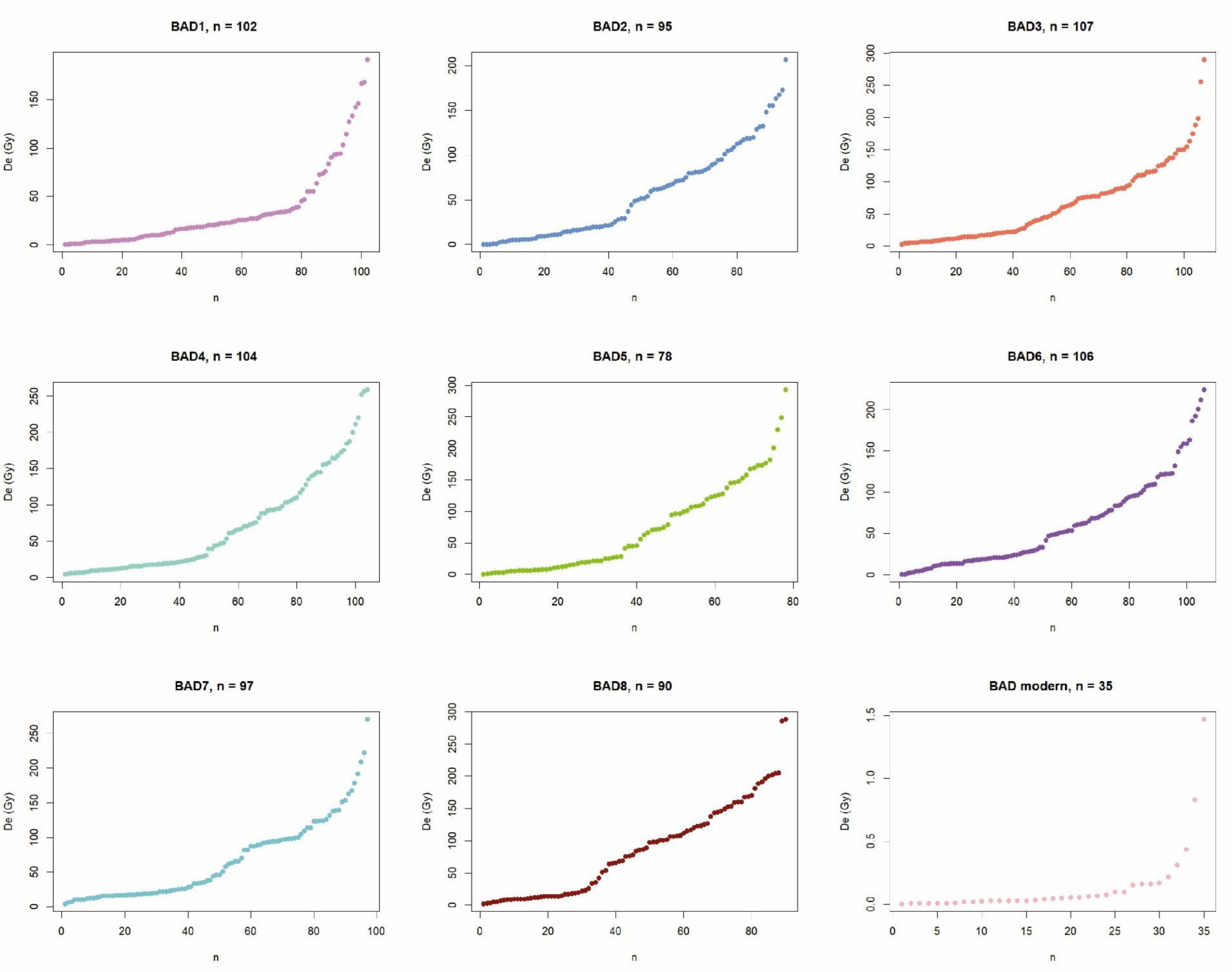

Figure S12. Distribution of D_e values.

Table S5. FMM results obtained on the Baden-Baden 2 samples using the σ_b that gives the lowest BIC score. The dose component and error, with the relative proportion are provided for each component (numbered C1, C2, and C3). The main component is shown in **bold**.

	BAD1	BAD2	BAD3	BAD4	BAD5	BAD6	BAD7	BAD8
Dose C1 (Gy)	3.27	4.90	12.73	15.18	6.333	4.73	18.20	2.60
± C1 (Gy)	0.40	0.92	1.15	1.14	0.92	1.13	1.25	0.89
Proportion C1 (%)	0.27	0.17	0.42	0.49	0.25	0.08	0.49	0.04
Dose C2 (Gy)	**21.90**	16.96	**88.05**	**108.47**	22.09	19.44	**103.13**	13.17
± C2 (Gy)	**1.80**	1.72	**6.65**	**7.89**	3.43	1.69	**7.08**	1.19
Proportion C2 (%)	**0.55**	0.31	**0.58**	**0.51**	0.25	0.4	**0.51**	0.34
Dose C3 (Gy)	87.42	**90.32**			**117.3**	**89.36**		**115.77**
± C3 (Gy)	13.24	**5.24**			**9.12**	**5.88**		**6.97**
Proportion C3 (%)	0.18	**0.53**			**0.51**	**0.52**		**0.63**
**σ_b**	**0.45**	**0.35**	**0.50**	**0.45**	**0.40**	**0.40**	**0.40**	**0.40**

Table S6. Breakdown of blanks by lithic raw material.

	Hornfels	Dolerite	Chert	Quartzite	Quartz	Syenite	Banded Ironstone	Total
U5-12, L4	27		1	1	2			31
U5-12, L5	35						1	36
U5-12, L6	54	1						55
U5-12, L7	7							7
U1-2, L3	12							12
U1-2, L4	22							22
U1-2, L5	1					5		6
U3, L2	64	1						65
Surface	222	11						233
Total	444	13	1	1	2	5	1	467

Table S7. Butt preparation of lithic blanks.

	Plane	Dihedral	facetted	*Ôté*	Siret	Punctiform	Semi-cort.	Cortical	Indet/Broken	Total
U5-12, L4	4	8	4			2			13	31
U5-12, L5	7	7	4	1	1	3	1		12	36
U5-12, L6	13	12	7	3		3			17	55
U5-12, L7	2		2						3	7
U1-2, L3	2		4	1	1				4	12
U1-2, L4	7	3	3	1					8	22
U1-2, L5	1	2	2						1	6
U3, L2	8	14	7		1	2			33	65
Surface	41	45	27	8	1	4	1	2	104	233
Total	85	91	60	14	4	14	2	2	195	467

Table S8. Distribution of modalities observed in blanks.

	Unipolar	Uni-conv.	Bipolar	Centripetal	Orthogonal	Divergent	Indet.	Total
U5-12, L4	3	3	3	1	2	1	18	31
U5-12, L5	5	10	5	2	2		11	36
U5-12, L6	7	9	10	6	8	3	12	55
U5-12, L7	2	2					3	7
U1-2, L3	2	5	3		1		2	12
U1-2, L4	7	2			6	1	6	22
U1-2, L5		1	1	2			2	6
U3, L2	23	12	9	2	6	1	12	65
Surface	49	42	40	29	14	4	55	233
Total	98	86	70	42	39	10	121	467

Table S9. FMM results obtained on the Baden-Baden 2 samples using $\sigma_b = 0.20$. The dose component and error, with the relative proportion are provided for each component (numbered C1, C2, C3, C4, and C5) obtained for the lowest BIC score. The main component is shown in **bold**.

	BAD1	BAD2	BAD3	BAD4	BAD5	BAD6	BAD7	BAD8
Dose C1 (Gy)	1.22	6.28	6.65	9.43	2.62	1.87	9.16	2.59
± C1 (Gy)	0.21	0.60	0.58	0.74	0.73	1.38	1.37	0.56
Proportion C1 (%)	0.07	0.22	0.15	0.2	0.05	0.02	0.08	0.04
Dose C2 (Gy)	4.06	19.34	16.84	**19.97**	7.51	5.71	18.08	10.95
± C2 (Gy)	0.31	1.30	1.02	**1.22**	0.67	0.83	1.38	1
Proportion C2 (%)	0.19	0.26	0.25	**0.28**	0.21	0.08	0.32	0.23
Dose C3 (Gy)	13.31	**92.78**	38.11	71.01	20.9	**19.7**	36.1	21.01
± C3 (Gy)	1.31	**3.48**	4.45	6.21	1.64	**0.83**	4.23	3.54
Proportion C3 (%)	0.20	**0.52**	0.1	0.25	0.2	**0.37**	0.14	0.1
Dose C4 (Gy)	**28.53**		**78.38**	154.51	63.21	64.17	**101.74**	73.44
± C4 (Gy)	**2.01**		**5.78**	12.06	6.64	5.47	**5.61**	9.76
Proportion C4 (%)	**0.36**		**0.30**	0.27	0.16	0.28	**0.41**	0.21
Dose C5 (Gy)	94.71		143.11		**140.95**	126.22	195.19	**144.92**
± C5 (Gy)	7.06		11.81		**8.14**	11.48	42.25	**10.58**
Proportion C5 (%)	0.17		0.20		**0.38**	0.25	0.06	**0.42**

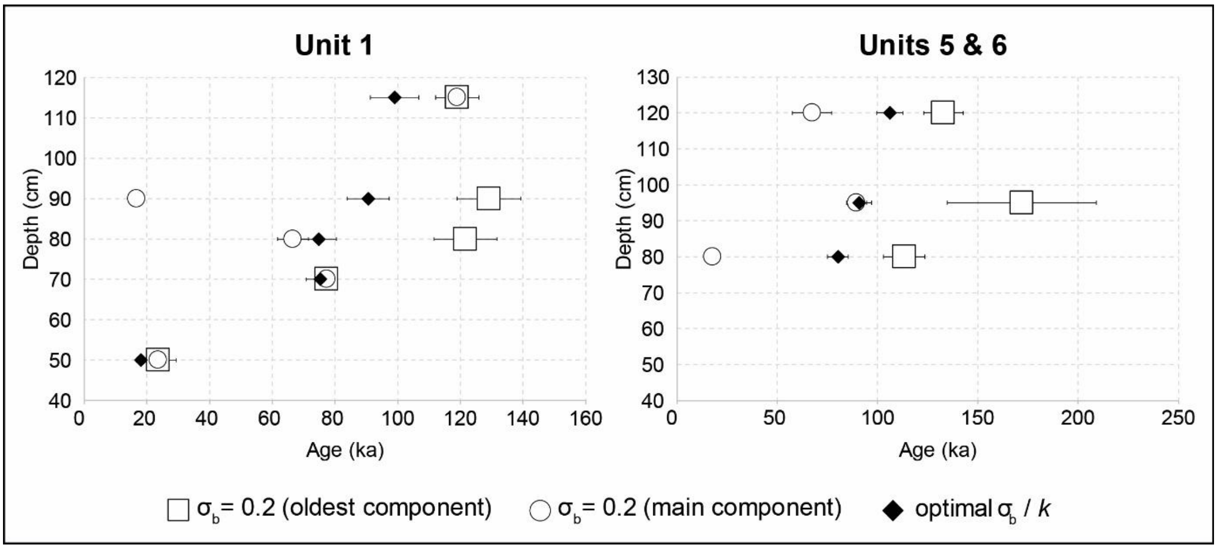

Figure S13. Graphic representation of ages as a function of depth for Unit 1 (left) and Units 5 & 6 (right). Circles: fixed $\sigma_b = 0.20$ using the main component; squares: fixed $\sigma_b = 0.20$ using the oldest component for BAD2 to BAD6, and the main component for BAD1; diamonds: optimal σ_b and k combination for the common dispersion (σ_b) using the main component. For some of the younger ages, the error bars are not visible due to the scale.

References

1 Stoops, G. *Guidelines for Analysis and Description of Soil and Regolith Thin Sections*. Second edition (Wiley, 2021). https://doi.org/10.1002/9780891189763

2 Wintle, A. G. & Murray, A. S. A review of quartz optically stimulated luminescence characteristics and their relevance in single-aliquot regeneration dating protocols. *Radiation Measurements* **41**, 369-391 (2006). https://doi.org/10.1016/j.radmeas.2005.11.001
